# The role of FOXO4/NFAT2 signaling pathway in dysfunction of human coronary endothelial cells and inflammatory infiltration of vasculitis in Kawasaki disease

**DOI:** 10.3389/fimmu.2022.1090056

**Published:** 2023-01-09

**Authors:** Hongbiao Huang, Jinfeng Dong, Jiaqi Jiang, Fang Yang, Yiming Zheng, Shuhui Wang, Nana Wang, Jin Ma, Miao Hou, Yueyue Ding, Lijun Meng, Wenyu Zhuo, Daoping Yang, Weiguo Qian, Qiaobin Chen, Guoping You, Guanghui Qian, Lei Gu, Haitao Lv

**Affiliations:** ^1^ Department of Pediatrics, Institute of Pediatric Research, Children’s Hospital of Soochow University, Suzhou, Jiangsu, China; ^2^ Department of Pediatrics, Fujian Provincial Hospital, Fujian Provincial Clinical College of Fujian Medical University, Fuzhou, Fujian, China; ^3^ Epigenetics Laboratory, Max Planck Institute for Heart and Lung Research, Bad Nauheim, Germany; ^4^ Department of Hematology, the First Affiliated Hospital of Fujian Medical University, Fuzhou, Fujian, China; ^5^ Department of Hematology, Children’s Hospital of Soochow University, Suzhou, China; ^6^ Department of Emergency, Fujian Provincial Hospital, Fujian Provincial Clinical College of Fujian Medical University, Fuzhou, Fujian, China; ^7^ Cardiopulmonary Institute (CPI), Bad Nauheim, Germany

**Keywords:** Kawasaki disease, FOXO4, Ca+/NFAT pathway, transcription factor, vasculitis

## Abstract

**Aims:**

The Ca+/NFAT (Nuclear factor of activated T cells) signaling pathway activation is implicated in the pathogenesis of Kawasaki disease (KD); however, we lack detailed information regarding the regulatory network involved in the human coronary endothelial cell dysfunction and cardiovascular lesion development. Herein, we aimed to use mouse and endothelial cell models of KD vasculitis *in vivo* and *in vitro* to characterize the regulatory network of NFAT pathway in KD.

**Methods and Results:**

Among the NFAT gene family, *NFAT2* showed the strongest transcriptional activity in peripheral blood mononuclear cells (PBMCs) from patients with KD. Then, *NFAT2* overexpression and knockdown experiments in Human coronary artery endothelial cells (HCAECs) indicated that *NFAT2* overexpression disrupted endothelial cell homeostasis by regulation of adherens junctions, whereas its knockdown protected HCAECs from such dysfunction. Combined analysis using RNA-sequencing and transcription factor (TF) binding site analysis in the *NFAT2* promoter region predicted regulation by Forkhead box O4 (FOXO4). Western blotting, chromatin immunoprecipitation, and luciferase assays validated that *FOXO4* binds to the promoter and transcriptionally represses *NFAT2*. Moreover, *Foxo4* knockout increased the extent of inflamed vascular tissues in a mouse model of KD vasculitis. Functional experiments showed that inhibition NFAT2 relieved *Foxo4* knockout exaggerated vasculitis *in vivo*.

**Conclusions:**

Our findings revealed the FOXO4/NFAT2 axis as a vital pathway in the progression of KD that is associated with endothelial cell homeostasis and cardiovascular inflammation development.

## 1 Introduction

Kawasaki disease (KD) is an acute vasculitis that is self−limiting and affects children. In developed countries, KD represents the most common cause of childhood acquired heart disease (1). Serious complications include coronary artery disease, which is closely related to the incidence of cardiovascular disease, especially coronary heart disease in adulthood (2), thus, a deeper mechanistic understanding of KD is required.

The incidence of KD differs among ethnicities, thus research linking genetic background to disease susceptibility has led to improved clinical trials (3). The only signaling pathway mentioned in the Genetics section of 2017 AHA guidelines as being related to clinical treatment is the Nuclear factor of activated T cells (NFAT) pathway (3). NFAT signaling affects immune cells and endothelial cells. The reasons for these phenotypes are the expression of downstream cytokines and adhesion proteins (4, 5). Although our understanding of the relationship between NFAT signaling and KD has developed in recent decades, the detailed mechanisms of NFAT activation in KD remain unknown.

NFAT was first identified as a member of an inducible nuclear protein complex involving interleukin-2 (IL-2) in T cells (6). The NFAT transcription factor family includes five members. NFAT1 (NFATc2 or NFATp) was first identified in 1993 (7). Phosphatase calcineurin regulates NFAT2 (NFATc1 or NFATc) (8), and NFAT3~5 were also identified recently (9). Research showed that NFAT acts as a transcriptional activator in the nucleus during the development of KD. Recent research showed that NFAT signaling activation disturbs the homeostasis of human coronary artery endothelial cells (HCAECs) (4). However, the function of the NFAT pathway in HCAECs, particularly in causing KD-related vasculitis, has not been determined.

In the present study, to identify the NFAT family member with the strongest transcriptional activation in KD, their relative expression in peripheral blood mononuclear cells (PBMCs) from patients with KD and dual luciferase experiments were carried out. NFAT2 had the strongest transcriptional activation in the NFAT family, and overexpression and knockdown of *NFAT2* in HCAECs demonstrated its important role in HCAEC homeostasis *in vitro*. We also predicted that the upstream transcription factor of *NFAT2* is Forkhead box O4 (FOXO4), which binds to the *NFAT2* promoter region, as verified using chromatin immunoprecipitation-quantitative polymerase chain reaction (ChIP-qPCR).

Next, we developed a *Candida albicans* water-soluble fraction (CAWS)-induced KD vasculitis mouse model, allowing us to detect NFAT2 and FOXO4 expression in PBMCs and heart tissues. The specific NFAT inhibitory peptide (11arginine (R)-VIVIT) has been used to observe the pathological changes after suppressing NFAT signaling (10). Moreover, by generating *Foxo4* knockout mice combined with (11R)-VIVIT, we demonstrated that FOXO4 is a negative regulator in CAWS-induced KD vasculitis and NFAT2 is a positive regulator. Blocking NFAT signaling reduced the severity of KD vasculitis-associated vascular inflammation in wild-type (WT) mice and *Foxo4* knockout mice. Therefore, we identified that NFAT2 promotes KD and FOXO4 transcriptionally represses *NFAT2* during the progression of KD vasculitis.

## 2 Methods

### 2.1 Sampling of human blood

The study was carried out following the tenets of the Declaration of Helsinki and the Ethics Committee of Soochow University Affiliated Children’s Hospital approved the study (Suzhou, China; approval no. 2020CS075). The Ethics Committee informed all the participants and their parents about the study details, who then provided written informed consent. Details about Sampling human blood are provided in the **Supplemental material**.

### 2.2 Genetically engineered mice

Details about genetically engineered mice are provided in the **Supplemental material**.

### 2.3 Preparation of CAWS

The CAWS was prepared from *Candida albicans* strain NBRC1385 using previously described methods (11, 12) and the details are provided in the **Supplemental material**.

### 2.4 CAWS-induced vasculitis in mouse model

All animal experiments were carried out following the Guide for the Care and Use of Laboratory Animals of the China National Institutes of Health, and the Animal Care and Use Committee of Soochow University approved the experiments (approval number: SUDA20220906A01). Details about the preparation of the genetically engineered mice are provided in the **Supplemental material**.

### 2.5 Histology and immunohistochemical staining

The sections were stained using hematoxylin and eosin (HE) and elastic van Gieson (EVG) staining as described previously (13). The severity of inflammatory infiltration was evaluated using heart vessel inflammation scores (14). The immunohistochemical quantification usied modified H-scores (15) and details are provided in the **Supplemental material**.

### 2.6 Immunofluorescence staining

More detailed descriptions about the experiments are provided in the **Supplemental material**.

### 2.7 Cell culture

Detailed descriptions of the cell culture conditions for HCAECs cells are provided in the **Supplemental Information**.

### 2.8 FOXO4 knockdown, FOXO4 overexpression, NFAT2 knockdown and NFAT2 overexpression in HCAEC cells

We packaged the lentiviruses according to a previously described method (16) and the details are provided in the **Supplemental material**.

### 2.9 Stimulation of cultured HCAECs with tumor necrosis factor-α

Details about these experiments are provided in the **Supplemental material**.

### 2.10 RNA extraction and quantitative real-time reverse transcription PCR

Experimental and primer details are provided in the **Supplemental material**. The 2^−ΔΔCt^ method (17) was used to analyze the qRT-PCR data.

### 2.11 Western blotting

The details about the western blotting analysis are provided in **Supplemental material**.

### 2.12 Luciferase assay

The details about the Luciferase assay are provided in the **Supplemental material**.

### 2.13 Chromatin immunoprecipitation assay

The ChIP assay was carried out according to a previously described method (18) and is detailed in the **Supplemental material**.

### 2.14 RNA sequencing

The details about the RNA-seq experiment are provided in the **Supplemental material**.

### 2.15 Cell proliferation assays

The details about cell proliferation assays are provided in the **Supplemental material**.

### 2.16 Statistical analysis

The details about the statistical analysis are provided in the **Supplemental material**.

## 3 Results

### 3.1 NFAT2 was significantly elevated in PBMCs from patients with KD and in the TNFα-stimulated HCAEC model

**Figure 1:**
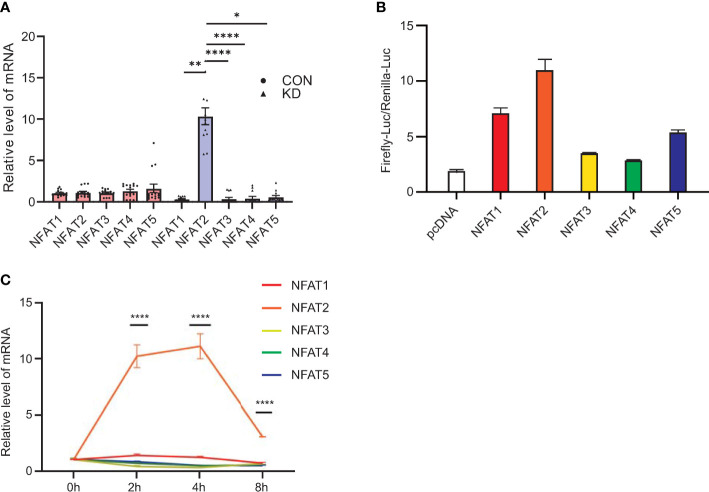
Levels of *NFAT2* mRNA increase during Kawasaki Disease progression. **(A)** qRT-PCR results for peripheral blood mononuclear cells (PBMCs) in blood samples from patients with Kawasaki Disease (n = 12) and healthy controls (n = 15). **(B)** 293T cells were transfected with control plasmid or *NFAT1*~*NFAT5*, together with NFAT_Luc and Renilla plasmids respectively. The cells were harvested 72 hours after transfection. The relative activity of NFAT-driven firefly luciferase activity was normalized to that of Renilla luciferase activity (Firefly-luc/Renilla-luc). **(C)** qRT-PCR results of NFAT family members in HCAEC that were stimulated by TNFα (40 ng/ml) at different timepoints (n = 3). Data are presented as the mean ± SEM. Quantitative data were analyzed using the Kruskal–Wallis test **(A)** and one-way ANOVA **(C)**, *P < 0.05, **P < 0.01, ****P < 0.0001. CON, control group; KD, Kawasaki Disease; NFAT, nuclear factor of activated T cells; HCAEC, Human coronary artery endothelial cells; ANOVA, analysis of variance.

qRT-PCR analysis of PBMCs revealed that compared with other members of the NFAT family, *NFAT2* mRNA levels were significantly upregulated in patients diagnosed with KD compared with that in the healthy controls (**Figure 1A**). The luciferase reporter assays showed that *NFAT2* had the strongest transcriptional activity among the NFAT family (**Figure 1B**). To further determine the critical roles of NFAT2 in KD, 40 ng/ml TNFα was used to stimulate HCAECs to mimic vasculitis *in vitro.* The dose of TNFα was determined by the expression of NFAT2 after HCAECs were stimulated by different doses (**Supplementary Figure 1A**). Over 0–8 hours of stimulation, *NFAT2* mRNA showed the highest upregulation compared with other members of the NFAT family (**Figure 1C**). Similarly, the RNA-seq results at different timepoints also showed that *NFAT2* expression increased most significantly compared with the TNFα stimulation group at zero hour (**Supplementary Figure 1B**). These findings clearly demonstrated upregulated *NFAT2* mRNA expression in patients and in vasculitis *in vitro*, suggesting that NFAT2 has an important function in the development of KD.

### 3.2 NFAT2 disrupted the homeostasis of endothelial cells by regulating adherens junctions

**Figure 2:**
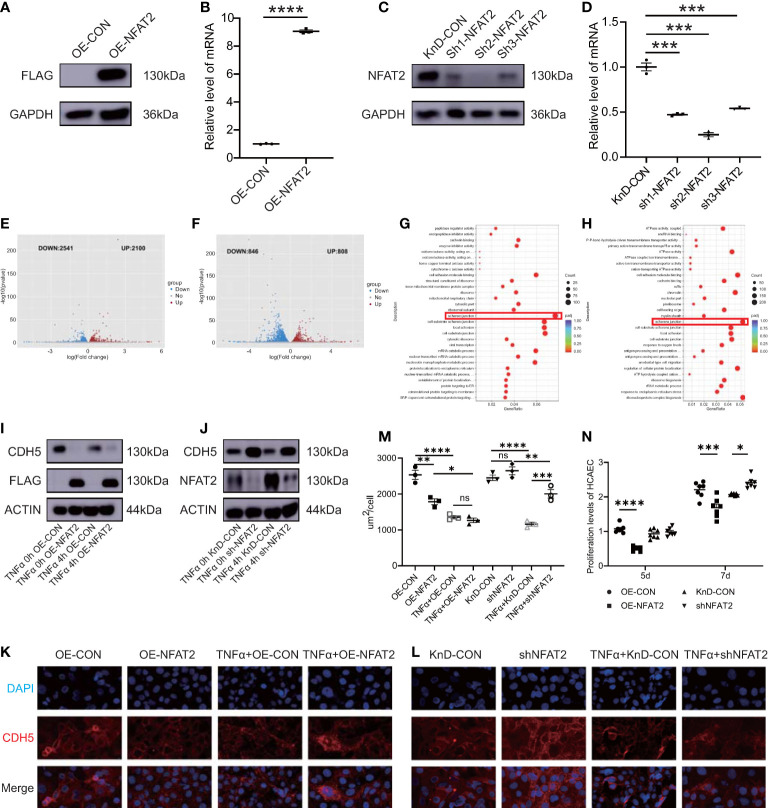
NFAT2 disrupted endothelial cell homeostasis. **(A, B)** At 7 days after transfection of *NFAT2* overexpressing lentiviruses ((OE-NFAT2) and their corresponding control (OE-CON), **(A)** NFAT2 protein levels were assayed using western blotting, with a loading control comprising GAPDH, and **(B)** *NFAT2* mRNA expression was assessed using qRT-PCR (n = 3). **(C, D)** At 7 days after transfection of lentiviruses for the knockdown *NFAT2* (sh1~sh3) and its corresponding controls (KnD-CON), **(C)** western blotting was used to assess NFAT2 protein levels, with a loading control comprising GAPDH, and **(D)** *NFAT2* mRNA expression was assessed using qRT-PCR (n = 3). **(E, F)** Volcano plot showing differentially expressed genes (DEGs) in response to *NFAT2* overexpression **(E)** or *NFAT2* knockdown **(F)**. Red dots represent upregulated genes; and blue dots represent downregulated genes. Gene Ontology (GO) functional enrichment analysis of DEGs related to *NFAT2* overexpression **(G)** and *NFAT2* knockdown **(H)**. **(I)** HCAECs transfected with *NFAT2* overexpressing lentiviruses (OE-NFAT2) and their corresponding control (OE-CON) were stimulated with/without TNFα (40 ng/ml) for 4 hours. CDH5 and NFAT2 protein levels were analyzed. β-Actin served as a loading control. **(J)** HCAECs transfected with *NFAT2* knockdown lentiviruses (shNFAT2) and their corresponding control (KnD-CON) were stimulated with/without TNFα (40 ng/ml) for 4 hours. CDH5 and NFAT2 protein levels were analyzed as described. β-Actin served as a loading control. **(K–M)** The expression of CDH5 was detected by immunofluorescence. *NFAT2* overexpression **(K)** and knockdown **(L)** vectors and their corresponding controls were transfected into HCAECs, respectively. After 7 days, cells were stimulated with/without TNFα (40 ng/ml) for 4 hours. The cells were immunostained with rabbit anti-CDH5, followed by staining with 594 goat anti-rabbit IgG (red). Cell nuclei were stained with DAPI (blue). The fluorescent images were captured **(K)**. The immunofluorescence area per cell in the different groups is shown **(M)** (n = 3). **(N)** HCAECs were transfected with *NFAT2* overexpression and knockdown vectors and their corresponding controls. After 5 days and 7 days, the proliferation of HCAECs were detected using a CCK8 assay (n = 7). Data are presented as the mean ± SEM. Quantitative data were analyzed using an unpaired t test (two-tailed) **(B, D)** and one-way ANOVA **(M, N)**, *P < 0.05, **P < 0.01, ***P < 0.001, ****P < 0.0001.

To investigate the function of NFAT2 in HCAECs, *NFAT2* overexpression (OE) and knockdown (KnD) lentiviruses were transfected into HCAECs and empty lentiviral vectors for overexpression (OE-CON) and knockdown (KnD-CON) were employed as the appropriate controls. *NFAT2* overexpression and knockdown in HCAECs were verified using western blotting and qRT-PCR (**Figures 2A–D**).

Next, we used RNA-Seq to investigate the mechanism of NFAT2 in HCAECs. Differentially expressed genes (DEGs) in the RNA-Seq data were identified using mRNA clustering and map plotting, with criteria of an absolute value log2 FoldChange > 0 and an adjusted p < 0.05. Overexpression of *NFAT2* resulted in the upregulation of 2100 genes and the downregulation of 2541 genes (**Figure 2E**). Knockdown of *NFAT2* resulted in the upregulation of 808 genes and the downregulation of 846 genes in HCAECs (**Figure 2F**). Gene ontology (GO) analysis was then used to functionally annotate the DEGs. The GO results indicated that the genes regulated by NFAT2 were involved in the regulation of adherens junctions, such as cell adhesion molecule binding and cell-substrate junction (**Figures 2G, H**).

To confirm the GO results, we chose the classical molecule Cadherin 5 (CDH5) to carry out an adherens junction experiment in HCAECs (19, 20). NFAT2 negatively regulated CDH5 expression in both the overexpression and knockdown group. The protein level of CDH5 decreased after TNFα stimulation, regardless of whether NFAT2 was overexpressed or not (**Figures 2I, J**). Consistently, immunofluorescence staining for CDH5 showed that overexpression of *NFAT2* notably disrupted CDH5 formation. Conversely, knockdown of *NFAT2* promoted the formation of CDH5. Regardless of the expression level of NFAT2, CDH5 levels were decreased in TNFα-stimulated cells compared with that in TNFα non-treated HCAECs (**Figures 2K–M**).

We also evaluated HCAEC proliferation using Counting Kit-8(CCK8) assays (4). The results showed that the proliferation of HCAECs in the OE-NFAT2 group at 5 and 7 days was significantly lower than that in the OE-CON group. In the KnD-NFAT2 group, HCAEC proliferation was not statistically significant at 5 days, but was significantly higher than that in the KnD-CON group at 7 days (**Figure 2N**). Collectively, the data indicated that NFAT2 has a vital function in the homeostasis of HCAECs.

### 3.3 FOXO4 negatively regulates NFAT2 in HCAECs

**Figure 3:**
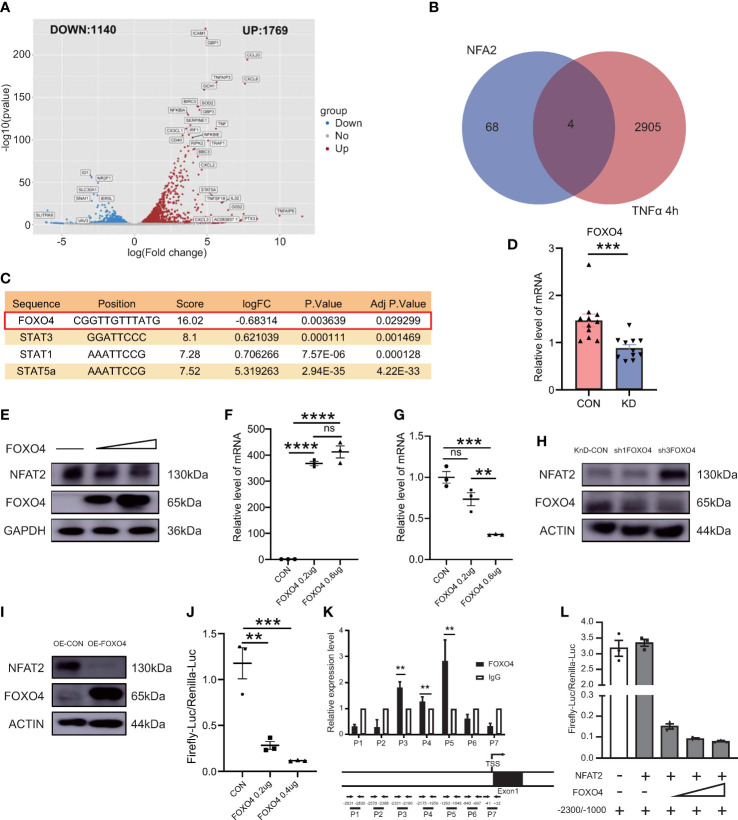
FOXO4 negatively regulates *NFAT2* by inhibiting its transcription binding to its promoter *in vitro*. **(A)** Volcano plot map of differentially expressed genes (DEGs) in HCAECs stimulated by TNFα (40ng/ml) for 4 hours. Red dots, upregulated genes; blue dots, downregulated genes. **(B)** Venn diagram displaying the overlapping genes between the DEGs in **(B)** and target genes predicted to have NFAT2 binding sites in their promoter or transcription start site according to the LASAGNA-Search 2.0 database. **(C)** Detailed information for the four overlapping genes. **(D)** *FOXO4* mRNA expression in peripheral blood mononuclear cells (PBMCs) in blood samples from patients with Kawasaki Disease (n = 11) and healthy controls (n = 11). **(E)** Immunoblotting analysis of NFAT2 levels in 293T cells transfected with the empty vector or an increasing amount of FOXO4 plasmid. **(F, G)** qRT-PCR results of 293T cells transfected with empty vector or an increasing amount of FOXO4 plasmid. mRNA expression of *FOXO4* ***(*F*)*** and *NFAT2* **(G)**. **(H–I)** HCAECs were transfected with *FOXO4* overexpression (OE-FOXO4) **(H)** and knockdown (shFOXO4) **(I)** lentiviruses and their corresponding controls for 7 days. FOXO4 and NFAT2 levels were analyzed. The loading control was β-Actin. **(J)** 293T cells were transfected with the control plasmid or increasing amounts of the FOXO4 plasmid, together with NFAT_Luc and Renilla plasmids, respectively. The cells were harvested at 72 hours after transfection. The relative NFAT-driven firefly luciferase activity was normalized to that of Renilla luciferase (Firefly-luc/Renilla-luc). **(K)** HCAECs transfected with *FOXO4* overexpressing lentiviruses were subjected to a ChIP assay. Real-time PCR with the indicated primers was used to assess the abundance of gene fragments in the input and immunoprecipitates. The upper image shows the *NFAT2* gene expression and the lower image shows the locations of primers for the ChIP assay. **(L)** The Firefly-luc/Renilla-luc in 293T cells transfected with the indicated plasmids. The location of the transcription start site in *NFAT2* was set as 0. Therefore, −2300 and −1000 indicate the upstream 1000~2300 bp fragments, respectively. IgG, immunoglobulin **(G)** Data are presented as the mean ± SEM. Quantitative data were analyzed using the Mann–Whitney test (two-tailed) **(D)** and one-way ANOVA **(F, G**, **J)**, **P < 0.01, ***P < 0.001, ****P < 0.0001.

Next, we investigated the regulation of NFAT2 *in vitro*. We identified genes with variable expression in the *in vitro* vasculitis model that could interact with the *NFAT2* promoter region as possible NFAT2 regulators. The RNA-seq analysis was carried out between HCAECs stimulated with TNFα for 4 h and unstimulated cells to identify DEGs (**Figure 3A**). The LASAGNA-Search 2.0 database was used to predict proteins that can interact with the promoter region of *NFAT2* (21) (**Supplementary Figure 1C**). Among the DEGs that were altered in the *in vitro* vasculitis model, four encoded proteins that might bind to the *NFAT2* promoter (**Figures 3B, C**). Among them, FOXO4 had highest prediction score. In addition, during TNFα stimulation of HCAECs for different times, *FOXO4* mRNA expression decreased significantly after 4 h (**Supplementary Figures 1B, D**).

To observe the expression of *FOXO4* in immune cells, we detected the mRNA expression of *FOXO4* in PBMCs from the healthy control group and patients with KD. Compared with that in the control group, the KD group had lower *FOXO4* expression in PBMCs (**Figure 3D**).

To identify the relationship between FOXO4 and NFAT2, several experiments were carried out *in vitro*. First, increasing amounts of the *FOXO4* overexpression plasmid were transfected into 293T cells. The immunoblotting results showed that *FOXO4* overexpression decreased the endogenous NFAT2 protein level in 293T cells. The increased amounts of transfected *FOXO4* led to a dose-dependent decrease in the levels of NFAT (**Figure 3E**). Consistent with protein level, *NFAT2* mRNA expression decreased gradually in cells overexpressing *FOXO4* (**Figures 3F, G**), suggesting that *FOXO4* regulates *NFAT2* at the mRNA level. We then showed that *FOXO4* overexpression decreased the protein level of NFAT2, whereas *FOXO4* knockdown increased it in HCAECs (**Figures 3H, I**). It is very difficult to transfect multiple plasmids into HCAECs at the same time; therefore, we used 293T cells for the luciferase assays, similar to previous research (22). Luciferase reporter assays showed that *FOXO4* overexpression inhibited the activation of the *NFAT2* reporter (**Figure 3J**), suggesting that FOXO4 regulates the transcription of *NFAT2*.

To further determine whether FOXO4 directly regulates *NFAT2* in HCAECs, we carried out a ChIP assay in HCAECs transfected with FLAG-tagged FOXO4 lentivirus to identify the binding regions. Primers were designed to amplify various genomic fragments upstream of the transcription start site of *NFAT2*. Real-time PCR assays of the immunoprecipitated DNA revealed that the P3~5 (−2331/−1049 bp) fragments had the highest enrichment (**Figure 3K**). Other fragments were not enriched compared with the immunoglobulin G control (**Figure 3K**). According to the luciferase activities, FOXO4 repressed both the basal *NFAT2* transcription and that driven by the −2300/−1000 fragment in a dose-dependent manner (**Figure 3L**). Thus, these results identified FOXO4 as a transcriptional repressor of *NFAT2* that physically binds to the −2300/−1000 region of its promoter.

### 3.4 FOXO4 stabilized the homeostasis of endothelial cells

**Figure 4:**
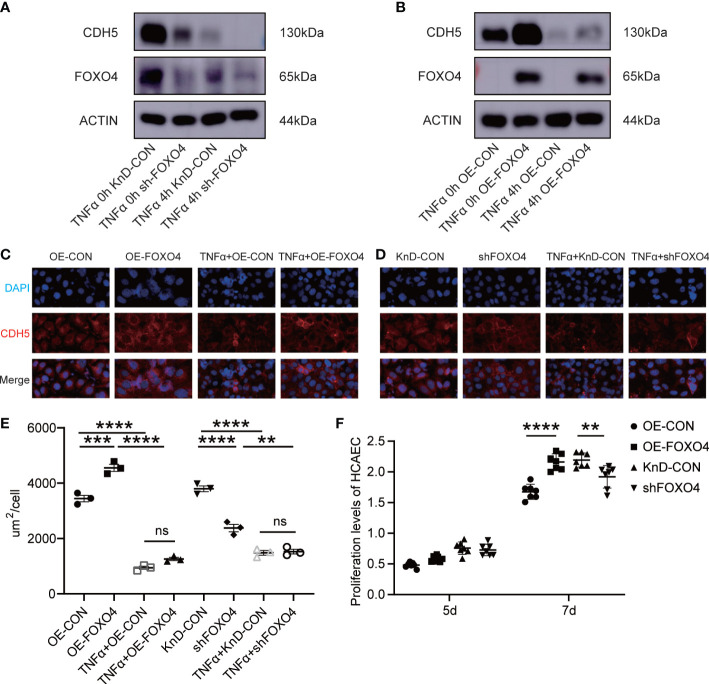
FOXO4 stabilized endothelial cell homeostasis *in vitro*. **(A)** HCAECs transfected with lentiviruses to knockdown *FOXO4* lentiviral (shFOXO4) and their corresponding controls (KnD-CON) were stimulated with/without TNFα (40 ng/ml) for 4 hours. CDH5 and FOXO4 levels were analyzed. The loading control was β-Actin. **(B)** HCAECs transfected with lentiviruses overexpressing *FOXO4* (OE-FOXO4) and their corresponding controls (OE-CON) were stimulated with/without TNFα (40 ng/ml) for 4 hours. CDH5 and NFAT2 protein level were analyzed. The loading control was β-Actin. **(C-E)** CDH5 expression was detected using immunofluorescence. *FOXO4* overexpression **(C)** and knockdown **(D)** vectors and their corresponding controls were transfected into HCAECs, respectively. After 7 days, cells were stimulated with/without TNFα (40 ng/ml) for 4 hours. The cells were then immunostained with rabbit anti-CDH5, followed by staining with 594 goat anti-rabbit IgG (red). Cell nuclei were stained with DAPI (blue) and the fluorescent images were captured. The immunofluorescence area per cell in the different groups (n = 3) **(E)**. **(F)** FOXO4 overexpression and knockdown vectors and their corresponding controls were transfected into HCAECs, respectively. After 5 days and 7 days, CCKL8 assays were used to detect HCAEC proliferation (n = 7). Data are presented as the mean ± SEM. Quantitative data were analyzed using one-way ANOVA **(E, F)**, **P < 0.01, ***P < 0.001, ****P < 0.0001.

Next, we investigated if FOXO4 has opposite functions to those of NFAT2. *FOXO4* overexpression (OE) and knockdown (KnD) lentiviruses were transfected into HCAECs. The protein level of CDH5 decreased after TNFα stimulation regardless of *FOXO4* overexpression (**Figures 4A, B**). Immunofluorescence staining for CDH5 indicated that *FOXO4* overexpression promoted CDH5 formation at intercellular borders, which was consistent with the western blotting results. TNFα-stimulated HCAECs showed lower CDH5 levels than TNFα non-treated HCAECs (**Figures 4C–E**).

The proliferation of HCAECs in the OE-FOXO4 group was significantly higher than that in the OE-CON group and proliferation in the KnD-FOXO4 group was significantly lower than that in the KnD-CON group at 7 days (**Figure 4F**). These data strongly suggested that FOXO4 has the opposite function to NFAT2 in maintaining endothelial cell homeostasis.

### 3.5 The expression of NFAT2 is upregulated in CAWS-induced vasculitis

**Figure 5:**
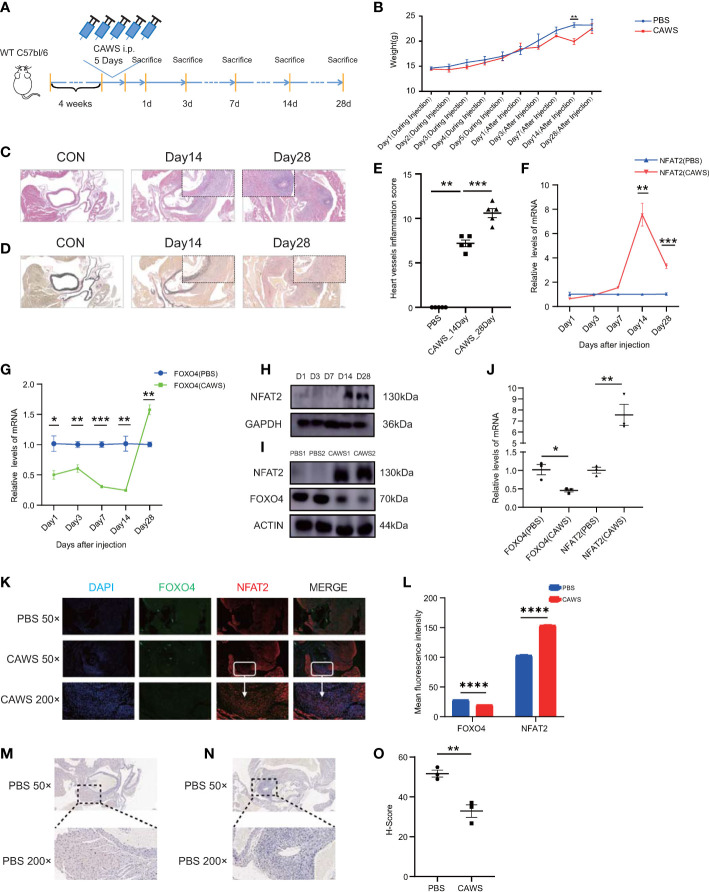
NFAT2 expression levels are upregulated during the progression of CAWS-induced vasculitis. **(A)** The protocol for constructing the CAWS-induced mouse model. **(B)** The change of weight during (n = 5) and after (n = 5) PBS/CAWS injection in mice. **(C, D)** At different timepoints the mouse were sacrificed, heart tissues were harvested, made into sections, and subjected to hematoxylin and eosin **(HE)** staining **(C)** and elastic van Gieson (EVG) staining **(D)**. Scale bars, 200 µm (50×) (Large) and 10 µm (200×) (Small). **(E)** Scores of heart vessel inflammation for WT mice injected with CAWS (n = 5). **(F–H)** NFAT expression was significantly upregulated at 14–28 days after CAWS injection, at the mRNA level in PBMCs (n = 3) **(F)** and at the protein level in heart tissues **(H)**, especially after 14 days. **(G)** The mRNA expression of *Foxo4* was almost opposite to that of *Nfat2* in PBMCs (n = 3). **(I, J)** NFAT expression was upregulated and that of FOXO4 was downregulated in heart tissues at 14 days after CAWS injection at both protein (n = 3) **(I)** and mRNA **(J)** levels. **(K)** Immunofluorescence imaging of FOXO4 (green) and NFAT2 (red) in heart sections from PBS/CAWS-injected 14 days WT group mice. Scale bars, 200 µm (50×) and 10 µm (200×). All images show DAPI staining of nuclei. **(L)** Mean fluorescence intensity of FOXO4 and NFAT2 in heart sections from PBS/CAWS-injected 14 days WT group mice. **(M–O)** Immunohistochemical staining for CDH5 in heart sections from PBS-injected **(M)** and CAWS-injected **(N)** WT group mice. CDH5 protein levels quantified using the H-score **(O)** (n = 3). Data are presented as the mean ± SEM. Quantitative data were analyzed using unpaired t tests (two-tailed) **(B, F, G, J, L, O)** and the Mann–Whitney test (two-tailed) **(E)** *P < 0.05, **P < 0.01, ***P < 0.001, ****P < 0.0001.

We further investigated the *in vivo* expression of NFAT2 and FOXO4. CAWS-induced vasculitis is widely used to study KD because the coronary artery lesions induced by CAWS are similar those of human KD (23). We tested different timepoints after CAWS injection to identify the most appropriate timepoint (**Figure 5A**). The weight of mice decreased slightly after the injection of CAWS, with the most obvious difference between the weight of the CAWS and control groups being observed at 14 days after CAWS injection (**Figure 5B**, **Supplementary Figure 2D**). HE and EVG staining indicated that perivascular inflammation began 14 days after CAWS injection, and the inflammatory infiltration was more obvious and the fibrous tissue damage was more serious at 28 days after CAWS injection (**Figures 5C–E**, **Supplementary Figure 2A–C**).

In PBMCs of CAWS injected mice, *Nfat2* expression increased significantly at 14 days after CAWS injection (**Figure 5F**) and *Foxo4* expression decreased (**Figure 5G**). Interestingly, the expression of *Foxo4* increased at 28 days (**Figure 5G** and **Supplementary Figure 2F–H**). In the heart tissue of the CAWS injected mice, the protein level of NFAT2 showed a similar phenomenon (**Figure 5H**). Therefore, we used 14 days after CAWS injection as the timepoint in the vasculitis model for follow-up studies. FOXO4 was downregulated at both the mRNA and protein level at 14 days after CAWS injection (**Figure 5I, J**). The LASAGNA-Search 2.0 database also predicted target proteins that bind the promoter of *Nfat2* in the mouse, including FOXO4 (**Supplementary Figure 2E**).

Next, we sought to determine whether the increase of NFAT2 was associated with the inflamed region from CAWS-injected heart tissue. Consequently, immunofluorescence was used to detect NFAT2 and FOXO4 expression in heart tissue sections from CAWS-injected mice. NFAT2 expression increased and FOXO4 decreased in the inflamed region of the heart tissue at 14 and 28 days after CAWS injection (**Figures 5K, L** and **Supplementary Figure 2F–H**). CDH5 expression decreased in the inflamed area, which was verified by western blotting (**Figures 5M–O**). Taken together, these data demonstrated that NFAT2 was upregulated and FOXO4 was downregulated in CAWS-induced heart tissues, especially in the inflamed region.

For better understand the phenotypic changes in inflamed region, we also performed immunofluorescence to detect classic immune cell infiltration. The results showed that macrophages (F4/80) and monocytes (CD14) infiltrated in the areas of high inflammation, which has been verified infiltrated in a KD-like mouse model abdominal aorta using single cell RNA-Seq (24) (**Supplement Figure 3 A–D**).

### 3.6 NFAT2 pharmacological blockade alleviates CAWS-induced KD vasculitis

**Figure 6:**
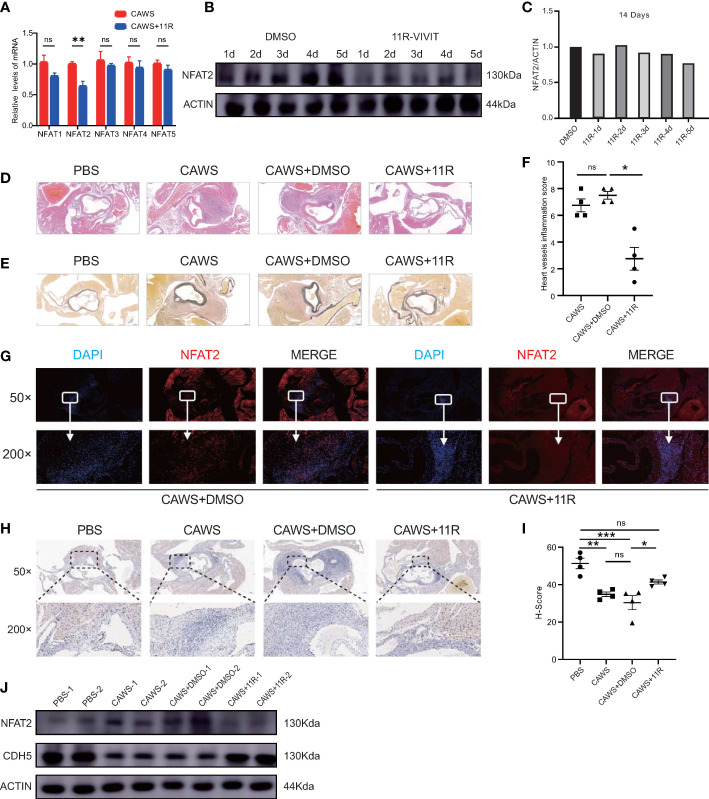
11R-VIVIT alleviated CAWS-induced KD vasculitis. **(A)** qRT-PCR results for NFAT family members in CAWS+11R-VIVIT injected heart tissue (n = 3). **(B, C)** Immunoblotting analysis of NFAT2 levels in mouse heart tissues from CAWS groups and mouse models injected with an increasing amount of 11R-VIVIT. The loading control comprised β-Actin **(B)**. NFAT2 levels in different groups quantified using ImageJ **(C)**. **(D–F)** Different groups the mouse were sacrificed and heart tissues were harvested, made into sections, and then subjected to hematoxylin and eosin (HE) staining **(D)** and elastic van Gieson (EVG) staining **(F)**. Heart vessel inflammation scores of WT mice in different groups was analyzed **(F)** (n = 4). **(G)** Immunofluorescent staining for NFAT2 (red) in heart sections from CAWS+DMSO/CAWS+11R-injected WT mice. All images show DAPI staining of nuclei. **(H)** immunohistochemical staining for CDH5 in heart sections from different groups. **(I)** CDH5 protein levels quantified by the H-score (n = 4). **(J)** Western blotting assessment of NFAT2 and CDH5 levels in different groups. The loading control comprised β-Actin. Scale bars, 200 µm (50×) and 10 µm (200×). Data are presented as the mean ± SEM. Quantitative data were analyzed using Kruskal–Wallis tests **(F)** and one-way ANOVA **(I)**, *P < 0.05, **P < 0.01, ***P < 0.001.

NFAT2 expression increased in the inflammatory regions and the Ca+/NFAT pathway plays an important role in KD (3); therefore, we used an NFAT2 inhibitor to determine whether it could inhibit inflammation in CAWS-induced KD vasculitis. The peptide 11R-VIVIT was used because it is a highly specific inhibitor of the NFAT signaling pathway. To observe the effect of 11R-VIVIT on the NFAT family, we detect the mRNA level of NFAT members in CAWS and 11R-VIVIT injected heart tissue. The results demonstrated the expression of *Nfat2* was downregulated most significantly among NFAT family members (**Figure 6A**). Compared with that in the CAWS+DMSO-injected control group, the NFAT2 protein level was significantly downregulated after 5 days of continuous 11R-VIVIT injection (**Figures 6B, C**).

To ascertain whether blocking NFAT2 directly would reduce CAWS-induced KD vasculitis, mice were administered with 11R-VIVIT for 5 consecutive days starting 1 h before CAWS injection. We found that 11R-VIVIT treatment significantly reduced CAWS-induced vasculitis (**Figures 6D–F**). NFAT2 expression also decreased in the inflamed region of the heart tissue at 14 days after 11E-VIVIT injection (**Figures 6G, J**). Treatment with 11R-VIVIT also increased CDH5 expression in the inflamed region of CAWS-injected heart tissue (**Figures 6H–J**). Taken together, our results showed that inhibition of NFAT2 using 11R-VIVIT partly prevented the development of CAWS-induced heart inflammation in mice.

### 3.7 NFAT2 acts as the downstream molecule of FOXO4 in CAWS-induced vasculitis

**Figure 7:**
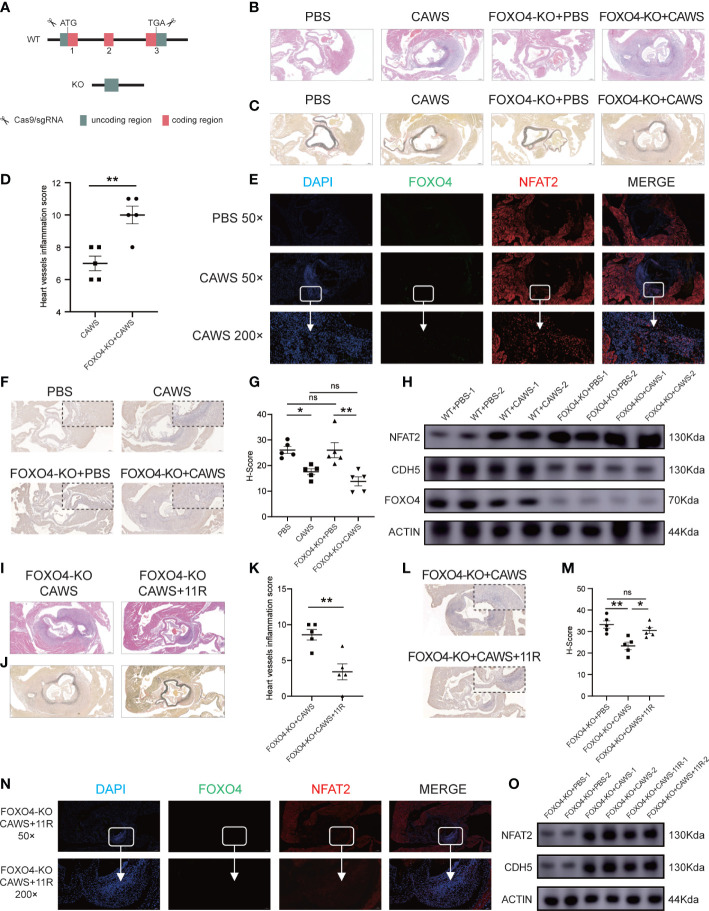
FOXO4-KO mice could exacerbate the vasculitis induced by CAWS, but the inflammation was relieved after blocking NFAT2. **(A)** The Cas9/gRNA method generated the FOXO4-KO mice. **(B–D)** Different groups of mice were sacrificed and heart tissues were harvested, made into section sections, and subjected to hematoxylin and eosin (HE)-staining **(B)** and elastic van Gieson (EVG) staining **(C)**. Heart vessel inflammation scores of FOXO4-KO and WT mice in different groups were analyzed **(D)** (n = 5). **(E)** Immunofluorescence staining for FOXO4 (green) and NFAT2 (red) in heart sections of the PBS/CAWS-injected FOXO4-KO group mice. All images show DAPI staining of nuclei. **(F)** Immunohistochemical staining for CDH5 in heart sections from four different groups. **(G)** The CDH5 protein level quantified by the H-score (n = 5). **(H)** Western blotting determination of NFAT2, FOXO4, and CDH5 protein levels in different groups. The loading control comprised β-Actin. Different groups of FOXO4-KO mouse were sacrificed, and heart tissues were harvested and made into sections, which were subjected to HE staining **(I)** and EVG staining **(J)** sections. **(K)** Heart vessel inflammation scores of FOXO4-KO+CAWS, and FOXO4-KO+CAWS+11R mice were analyzed (n = 5). **(L)** Immunohistochemical staining for CDH5 in heart sections from different FOXO4-KO groups. **(M)** CDH5 protein levels quantified by the H-score (n = 5). **(N)** Immunofluorescence staining for FOXO4 (green) and NFAT2 (red) in heart sections from FOXO4-KO+CAWS+11R-injected group mice. All images show DAPI staining of nuclei. **(O)** Western blotting analysis of NFAT2 and CDH5 levels in different FOXO4-KO mouse groups. The loading control comprised β-Actin. Scale bars, 200 µm (50×) and 10 µm (200×). Data are presented as the mean ± SEM. Quantitative data were analyzed using unpaired t tests (two-tailed) **(D, K)** and one-way ANOVA **(E, M)**, *P < 0.05, **P < 0.01.

To determine FOXO4’s *in vivo* functions, we bought the *Foxo4*-KO mouse from Cyagen Biosciences (designated as Foxo4^em1cyagen^). The *Foxo4* gene (NM_018789) is located on mouse chromosome X and comprises three exons. To produce the KO mouse, all three exons were targeted by Cas9/gRNA co-injection into fertilized eggs (**Figure 7A**). Loss of *Foxo4* in the *Foxo4*-KO mice was verified by DNA electrophoresis (**Supplementary Figure 4A**). Wild-type (WT) mice and Foxo4^em1cyagen^ mice were injected with PBS or CAWS, respectively. After 14 days of injection, heart tissue was processed for HE and EVG-staining for histological examination (**Supplementary Figure 4B**). Compared with that in the WT group, the Foxo4^em1cyagen^ group showed increased heart artery inflammation after CAWS-induced KD vasculitis. There were no significant differences after PBS injection in both the WT and Foxo4^em1cyagen^ groups (**Figures 7B–D**). NFAT2 expression increased in the CAWS-injected Foxo4^em1cyagen^ group compared with that in the PBS-injected Foxo4^em1cyagen^ group and the CAWS-injected WT group (**Figures 7E, H**, **Supplementary Figure 4C**). In contrast, CDH5 expression decreased significantly in the CAWS-injected Foxo4^em1cyagen^ group compared with that in PBS injection group (**Figures 7F–H**).

These results encouraged us to assess whether FOXO4 modulates CAWS-induced vasculitis through NFAT2. We carried out rescue experiments in the Foxo4^em1cyagen^ group by simultaneously injecting CAWS and inhibiting NFAT2 using 11R-VIVIT (**Supplementary Figure 4D**). We observed that injection of 11R-VIVIT in the CAWS-injected Foxo4^em1cyagen^ group partly alleviated heart artery inflammation and the heart vessel inflammation scores also decreased (**Figures 7I–K**). Furthermore, 11R-VIVIT partially increased the expression of CDH5 and decreased the expression of NFAT2 in the inflamed region of CAWS-induced heart tissue (**Figures 7L–O**, **Supplementary Figure 4E**). As a result, we concluded that knockout of *Foxo4* promotes inflammation in CAWS-induced KD vasculitis, at least in part, by activating the transcription of *Nfat2*.

## 4 Discussion

Initially, this study was prompted by the observation that transcription factor (TF) NFAT2 is upregulated in immune cells and stromal cells through NFAT signaling, a pathway associated with KD. Recent studies on KD showed that the formation of vasculitis is highly related to the infiltration of immune cells into stromal cells (24). To better understand the role of the NFAT signaling pathway, especially that of NFAT2, in KD progression, we conducted our research from three different aspects. First, we identified that NFAT2 has the strongest transcriptional activation among NFAT family member. Second, we used TNFα-stimulated HCAECs to simulate the infiltration of stromal cells in vasculitis. Third, we used a CAWS-induced mouse model of KD vasculitis to study the overall changes in heart tissue, including both immune cells and stromal cells. In this study, we identified a novel pathway comprising FOXO4/NFAT2. This pathway affects endothelial cell homeostasis *in vitro* and formation of CAWS-induced vasculitis *in vivo*. Our results showed that the downregulation of FOXO4 promoted NFAT2 expression, causing an imbalance in endothelial cell homeostasis and worsening of vascular inflammatory infiltration. Inhibition of *Nfat2* in *Foxo4*-KO mice reduced the level of inflammatory infiltration.

We found the NFAT2 expression was significantly elevated in immune cells (PBMCs) from patients with KD, similar to a previous study (25). Previous research also reported that NFAT inhibitors, such as cyclosporine, can prevent progression of inflammation in the arterial wall by blocking cytotoxic CD8+T cells infiltration into the arterial wall (26). This might represent the important impact of NFAT inhibitors KD patients’ PBMCs. This could be explained by the fact that the Ca+/NFAT signaling pathway is activated in the acute stage of KD, and NFAT2 is an important TF in this pathway. In addition, NFAT2 was upregulated in PBMCs from CAWS-induced mice. To date, there has been no report about CAWS directly activating the NFAT signaling pathway; however, higher production of proinflammatory cytokines, including TNFα and interleukin (IL)-1β, has been observed in the serum of CAWS-injected mice (27, 28). This might be the reasons why the NFAT pathway is activated and NFAT2 is upregulated.

As important stromal cells in heart tissue, HCAECs also have an important relationship with intravascular thrombosis (29), which is considered one of the most serious complications of KD (3). We selected HCAECs to study the mechanism of KD from the perspective of stromal cells. TNFα plays an importance role in KD, and was significantly elevated in patients’ plasma; therefore, anti-TNF-α therapy has been a common treatment option for patients with intravenous immunoglobulin (IVIG) resistant KD (30–32). Consequently, we used TNFα to stimulate HCAECs to simulate the effect of inflammatory factors on stromal cells *in vitro*. Similar to previous research, NFAT2 expression increased significantly after TNFα stimulation (4). To better understand the changes in NFAT2 expression *in vivo*, we observed the expression of NFAT2 in heart tissue, especially in the inflamed areas, after CAWS-induced vasculitis. Those observations were similar to those made in previous research, in which stimulation by proinflammatory cytokines, such as TNFα, upregulated NFAT2 in both PBMCs and endothelial cells (4, 33). We also found that inhibiting NFAT2 expression using 11R-VIVIT could alleviate vascular inflammation. 11R-VIVIT is not a specific inhibitor of NFAT2. However, no specific inhibitor of NFAT2 is currently available. *NFAT2* shows the strongest transcriptional activity, thus most 11R-VIVIT studies have focused on NFAT2 rather than other members of NFAT family, as did our design (34, 35). 11R-VIVIT selectively interferes with the calcineurin-NFAT2 interaction without altering the calcineurin phosphatase activity *in vivo* and *in vitro* (36–38).

We also found that an increase of NFAT2 in endothelial cells decreased the function of intercellular junctions *via* CDH5. *In vitro*, similar to previous experiments using human umbilical vein endothelial cells, CDH5 expression decreased significantly after being stimulated by proinflammatory factors (39). *In vivo*, cardiac ischemic injury, cardiac fibrosis, and even occlusion formation were also observed in *Cdh5*-KO mice (39, 40). These changes are similar to the cardiovascular manifestations of KD. Moreover, in acute KD, dysregulation of endothelial cell homeostasis probably affects aneurysm formation and vascular wall injury (4). These might be one of reasons why suppressing the Ca+/NFAT signaling pathway can reduce coronary artery lesions in KD.

Mammals have four FOXO TFs: FOXO1, FOXO3a, FOXO4, and FOXO6 (41). FOXO4 is mainly involved in cell cycle arrest, apoptosis, and muscle homeostasis (42). To date, there has been no research on the role or mechanism of FOXO4 in KD. However, previous studies provided several possibilities: 1) Inflammatory cytokine expression. A previous study reported that FOXO4 represses the expression of inflammatory cytokines, such as TNFα and IL-1β, which have vital functions in the mechanism of KD (43). 2) Preventing aortic aneurysm formation. Blocking the nuclear translocation of FOXO4 stimulated aortic aneurysm formation (44). Coronary or aortic aneurysms are important complications of KD; therefore; FOXO4 might have a protective role in the progress of KD.

At day 28 after CAWS injection, we found the mRNA and protein levels of FOXO4 were upregulated, for which there are two possible reasons. Firstly, it might suggest that the heart tissue is entering the subacute phase/recovery phase. Acute arteritis mainly involves immune cell infiltration, and subacute chronic arteritis in KD involves luminal myofibroblast proliferation (23). In our mouse model, at day 14, arteries are mainly infiltrated by immune cells, and their shape is normal. However, at day 28, luminal myofibroblasts obviously proliferate and vessels lose their original shape (**Figure 5D**), which indicated that vasculitis has entered the subacute phase. We found that FOXO4 might be a protector in the process of KD-like vasculitis, such that the level of FOXO4 could increase in the subacute or recovery phase. Secondly, FOXO4 might be uncoupled from *NFAT2* when the disease enters the subacute phage; but the mechanism of this phenotype needs to be further studied in the future. At day 28, the CAWS mice tended to regain the weight lost by day 14, perhaps for the same reason. When the mice entered into the subacute phase, their weight recovered gradually.

Interestingly, *in vivo*, the level of inflammation in CAWS-injected heart tissue in the *Foxo4*-KO group was more serious than that in the WT group. Previous studies using *Foxo4*-KO mice reported that the *Foxo4*-KO group produced more severe inflammatory infiltration (43, 45). *Foxo4*-KO immune cells would increase resident smooth muscle cell proliferation and endothelial cell dysfunction, which would further enhance inflammation and the formation of coronary/aorta vasculitis (45). Consistently, we demonstrated that the NFAT inhibitor, 11R-VIVIT, attenuated CAWS-induced inflammatory responses in *Foxo4*-KO heart tissue. This indicated that the FOXO4/NFAT2 signaling pathway functions not only in HCAECs, but also in mouse heart tissue.

There are several limitations of this study. First, we only examined PBMCs from patients with KD and health controls, and further study should focus on patients with KD with and without coronary artery lesions. Second, the mechanism by which FOXO4 is downregulated in different cells or tissues remains unknown. Third, although we demonstrated that FOXO4 inhibits *NFAT2* transcription by physically binding to a region of its promoter, the exact mechanism of this inhibition remains to be determined. For example, does FOXO4 represses *NFAT2* transcription by competitive inhibition of its activator and why does FOXO4 appear to be uncoupled from *Nfat2* when the mice entered the subacute phage. Fourth, 11R-VIVIT might affect other NFAT family members; therefore, we need a specific inhibitor of NFAT2 to improve our experiments in the future. These aspects will be examined in future studies.

In this study, NFAT2 was identified to have an important role in the Ca+/NFAT pathway. Moreover, partly through its negative regulation of NFAT2, FOXO4 functions as a transcriptional repressor to suppress vasculitis and maintain endothelial cell homeostasis, thereby controlling vasculitis in KD. The FOXO4/NFAT2 signaling pathway could be developed as a novel therapeutic target, and exploiting its related intrinsic inhibitory mechanisms could lead to novel therapies to prevent and treat KD.

## Data availability statement

The original contributions presented in the study are publicly available. This data can be found here: GEO, GSE210094.

## Ethics statement

The studies involving human participants were reviewed and approved by the Ethics Committee of Soochow University Affiliated Children’s Hospital (approval no. 2020CS075). Written informed consent to participate in this study was provided by the participants’ legal guardian/next of kin. The animal study was reviewed and approved by Animal Care and Use Committee of Soochow University.

## Author contributions

HH conceived and coordinated the project, analyzed the data, and wrote the paper. HH, JD, and JJ performed the majority of the experiments. YZ performed the qRT-PCR experiments. JD and SW carried out the transfection of lentivirus targeting FOXO4 and NFAT2 experiments. JD and JJ supported the ChIP-qPCR experiments. HH, JJ, YZ, and NW performed the animal experiments. YD, JM and MH help with the *in vivo* experiments. WZ, FY, LM, DY, GY, and QC provided critical ideas and comments for the NFAT2 and FOXO4 study, HL, HH, JD, DY, WQ and GQ discussed the results. HL and LG critically discussed the data and read and revised the manuscript. All authors contributed to the article and approved the submitted version.

## References

[1] Huang H Xu L Ding Y Qin J Huang C Li X. Bioinformatics identification of hub genes and signaling pathways regulated by intravenous immunoglobulin treatment in acute Kawasaki disease. Exp Ther Med (2021) 22(1):784. doi: 10.3892/etm.2021.10216 34055083 PMC8145699

[2] Fukazawa R Kobayashi J Ayusawa M Hamada H Miura M Mitani Y. Jcs/Jscs 2020 guideline on diagnosis and management of cardiovascular sequelae in Kawasaki disease. Circ J (2020) 84(8):1348–407. doi: 10.1253/circj.CJ-19-1094 32641591

[3] McCrindle BW Rowley AH Newburger JW Burns JC Bolger AF Gewitz M. Diagnosis, treatment, and long-term management of Kawasaki disease: A scientific statement for health professionals from the American heart association. Circulation (2017) 135(17):e927–99. doi: 10.1161/CIR.0000000000000484 28356445

[4] Wang Y Hu J Liu J Geng Z Tao Y Zheng F. The role of Ca(2+)/Nfat in dysfunction and inflammation of human coronary endothelial cells induced by sera from patients with Kawasaki disease. Sci Rep (2020) 10(1):4706. doi: 10.1038/s41598-020-61667-y 32170198 PMC7069934

[5] Onouchi Y Gunji T Burns JC Shimizu C Newburger JW Yashiro M. Itpkc functional polymorphism associated with Kawasaki disease susceptibility and formation of coronary artery aneurysms. Nat Genet (2008) 40(1):35–42. doi: 10.1038/ng.2007.59 18084290 PMC2876982

[6] Serfling E Barthelmäs R Pfeuffer I Schenk B Zarius S Swoboda R. Ubiquitous and lymphocyte-specific factors are involved in the induction of the mouse interleukin 2 gene in T lymphocytes. EMBO J (1989) 8(2):465–73. doi: 10.1002/j.1460-2075.1989.tb03399.x PMC400828 2542017

[7] McCaffrey PG Luo C Kerppola TK Jain J Badalian TM Ho AM. Isolation of the cyclosporin-sensitive T cell transcription factor nfatp. Science (1993) 262(5134):750–4. doi: 10.1126/science.8235597 8235597

[8] Northrop JP Ho SN Chen L Thomas DJ Timmerman LA Nolan GP. Nf-at components define a family of transcription factors targeted in T-cell activation. Nature (1994) 369(6480):497–502. doi: 10.1038/369497a0 8202141

[9] Vaeth M Feske S. Nfat control of immune function: New frontiers for an abiding trooper. F1000Research (2018) 7:260. doi: 10.12688/f1000research.13426.1 29568499 PMC5840618

[10] Aramburu J Yaffe MB López-Rodríguez C Cantley LC Hogan PG Rao A. Affinity-driven peptide selection of an nfat inhibitor more selective than cyclosporin a. Science (1999) 285(5436):2129–33. doi: 10.1126/science.285.5436.2129 10497131

[11] Stock AT Hansen JA Sleeman MA McKenzie BS Wicks IP. Gm-csf primes cardiac inflammation in a mouse model of Kawasaki diseasegm-csf triggers cardiac inflammation. J Exp Med (2016) 213(10):1983–98. doi: 10.1084/jem.20151853 PMC5030799 27595596

[12] Jia C Zhang J Chen H Zhuge Y Chen H Qian F. Endothelial cell pyroptosis plays an important role in Kawasaki disease *Via* Hmgb1/Rage/Cathespin b signaling pathway and Nlrp3 inflammasome activation. Cell Death Dis (2019) 10(10):778. doi: 10.1038/s41419-019-2021-3 31611559 PMC6791856

[13] Hamaoka-Okamoto A Suzuki C Yahata T Ikeda K Nagi-Miura N Ohno N. The involvement of the vasa vasorum in the development of vasculitis in animal model of Kawasaki disease. Pediatr Rheumatol (2014) 12(1):1–9. doi: 10.1186/1546-0096-12-12 PMC3986644 24678599

[14] Lee Y Schulte DJ Shimada K Chen S Crother TR Chiba N. Interleukin-1beta is crucial for the induction of coronary artery inflammation in a mouse model of Kawasaki disease. Circulation (2012) 125(12):1542–50. doi: 10.1161/CIRCULATIONAHA.111.072769 PMC3337219 22361326

[15] Paschalis A Sheehan B Riisnaes R Rodrigues DN Gurel B Bertan C. Prostate-specific membrane antigen heterogeneity and DNA repair defects in prostate cancer. Eur Urol (2019) 76(4):469–78. doi: 10.1016/j.eururo.2019.06.030 PMC6853166 31345636

[16] La Madrid AM Campbell N Smith S Cohn SL Salgia R. Targeting alk: A promising strategy for the treatment of non-small cell lung cancer, non-hodgkin’s lymphoma, and neuroblastoma. Targ Oncol (2012) 7(3):199–210. doi: 10.1007/s11523-012-0227-8 22968692

[17] Livak KJ Schmittgen TD. Analysis of relative gene expression data using real-time quantitative pcr and the 2– Δδct method. Methods (2001) 25(4):402–8. doi: 10.1006/meth.2001.1262 11846609

[18] Chen Y-L Li X-L Li G Tao Y-F Zhuo R Cao H-B. Brd4 inhibitor Gne987 exerts anti-cancer effects by targeting super-enhancers in neuroblastoma. Cell Biosci (2022) 12(1):1–20. doi: 10.1186/s13578-022-00769-8 35303940 PMC8932231

[19] Skaria T Bachli E Schoedon G. Wif1 prevents Wnt5a mediated Limk/Cfl phosphorylation and adherens junction disruption in human vascular endothelial cells. J Inflammation (Lond) (2017) 14:10. doi: 10.1186/s12950-017-0157-4 PMC5437570 28529460

[20] Bai S Wei Y Hou W Yao Y Zhu J Hu X. Orai-Igfbp3 signaling complex regulates high-glucose exposure-induced increased proliferation, permeability, and migration of human coronary artery endothelial cells. BMJ Open Diabetes Res Care (2020) 8(1):e001400. doi: 10.1136/bmjdrc-2020-001400 PMC7580052 33087338

[21] Lee C Huang C-H. Lasagna-search 2.0: Integrated transcription factor binding site search and visualization in a browser. Bioinformatics (2014) 30(13):1923–5. doi: 10.1093/bioinformatics/btu115 24578403

[22] Fang J Ji Y-X Zhang P Cheng L Chen Y Chen J. Hepatic Irf2bp2 mitigates nonalcoholic fatty liver disease by directly repressing the transcription of Atf3. Hepatology (2020) 71(5):1592–608. doi: 10.1002/hep.30950 31529495

[23] Noval Rivas M Arditi M. Kawasaki Disease: Pathophysiology and insights from mouse models. Nat Rev Rheumatol (2020) 16(7):391–405. doi: 10.1038/s41584-020-0426-0 32457494 PMC7250272

[24] Porritt RA Zemmour D Abe M Lee Y Narayanan M Carvalho TT. Nlrp3 inflammasome mediates immune-stromal interactions in vasculitis. Circ Res (2021) 129(9):e183–200. doi: 10.1161/CIRCRESAHA.121.319153 PMC8555446 34517723

[25] Lv YW Chen Y Lv HT Li X Tang YJ Qian WG. Kawasaki Disease Ox40-Ox40l axis acts as an upstream regulator of nfat signaling pathway. Pediatr Res (2019) 85(6):835–40. doi: 10.1038/s41390-019-0312-0 30723312

[26] Burns JC. Cyclosporine and coronary outcomes in Kawasaki disease. J Pediatr (2019) 210:239–42. doi: 10.1016/j.jpeds.2019.04.044 31234982

[27] Nakamura J Watanabe S Kimura H Kobayashi M Karasawa T Kamata R. Adeno-associated virus vector-mediated interleukin-10 induction prevents vascular inflammation in a murine model of Kawasaki disease. Sci Rep (2018) 8(1):7601. doi: 10.1038/s41598-018-25856-0 29765083 PMC5953966

[28] Zhang J Zhuge Y Rong X Ni C Niu C Wen Z. Protective roles of xijiao dihuang tang on coronary artery injury in Kawasaki disease. Cardiovasc Drugs Ther (2021). doi: 10.1007/s10557-021-07277-w 34665368

[29] Chin-Quee SL Hsu SH Nguyen-Ehrenreich KL Tai JT Abraham GM Pacetti SD. Endothelial cell recovery, acute thrombogenicity, and monocyte adhesion and activation on fluorinated copolymer and phosphorylcholine polymer stent coatings. Biomaterials (2010) 31(4):648–57. doi: 10.1016/j.biomaterials.2009.09.079 19822362

[30] Burns JC Mason WH Hauger SB Janai H Bastian JF Wohrley JD. Infliximab treatment for refractory Kawasaki syndrome. J Pediatr (2005) 146(5):662–7. doi: 10.1016/j.jpeds.2004.12.022 15870671

[31] Shimizu M Mizuta M Usami M Inoue N Sakakibara Y Yamada K. Clinical significance of serum soluble tnf receptor ii level and soluble tnf receptor Ii/I ratio as indicators of coronary artery lesion development in Kawasaki disease. Cytokine (2018) 108:168–72. doi: 10.1016/j.cyto.2018.03.037 29625336

[32] Yamaji N da Silva Lopes K Shoda T Ishitsuka K Kobayashi T Ota E. Tnf-alpha blockers for the treatment of Kawasaki disease in children. Cochrane Database Syst Rev (2019) 8:CD012448. doi: 10.1002/14651858.CD012448.pub2 31425625 PMC6953355

[33] Yokota K Sato K Miyazaki T Aizaki Y Tanaka S Sekikawa M. Characterization and function of tumor necrosis factor and Interleukin-6–induced osteoclasts in rheumatoid arthritis. Arthritis Rheumatol (2021) 73(7):1145–54. doi: 10.1002/art.41666 PMC8361923 33512089

[34] Xie Z-Y Dong W Zhang L Wang M-J Xiao Z-M Zhang Y-H. Nfat inhibitor 11r-vivit ameliorates mouse renal fibrosis after ischemia-Reperfusion-Induced acute kidney injury. Acta Pharmacol Sin (2022) 43(8):2081–93. doi: 10.1038/s41401-021-00833-y PMC9343462 34937917

[35] Liu F Zhu Z Mao Y Liu M Tang T Qiu S. Inhibition of titanium particle-induced osteoclastogenesis through inactivation of Nfatc1 by vivit peptide. Biomaterials (2009) 30(9):1756–62. doi: 10.1016/j.biomaterials.2008.12.018 19118894

[36] Dou C Zhang H Ke G Zhang L Lian Z Chen X. The kruppel-like factor 15-Nfatc1 axis ameliorates podocyte injury: A novel rationale for using glucocorticoids in proteinuria diseases. Clin Sci (Lond) (2020) 134(12):1305–18. doi: 10.1042/CS20200075 32478397

[37] Noguchi H Sugimoto K Miyagi-Shiohira C Nakashima Y Kobayashi N Saitoh I. Rcan-11r peptide provides immunosuppression for fully mismatched islet allografts in mice. Sci Rep (2017) 7(1):3043. doi: 10.1038/s41598-017-02934-3 28596584 PMC5465209

[38] Zhang L Li R Shi W Liang X Liu S Ye Z. Nfat2 inhibitor ameliorates diabetic nephropathy and podocyte injury in Db/Db mice. Br J Pharmacol (2013) 170(2):426–39. doi: 10.1111/bph.12292 PMC3834765 23826864

[39] Jordan NP Tingle SJ Shuttleworth VG Cooke K Redgrave RE Singh E. Mir-126-3p is dynamically regulated in endothelial-to-Mesenchymal transition during fibrosis. Int J Mol Sci (2021) 22(16):8629. doi: 10.3390/ijms22168629 34445337 PMC8395326

[40] Patel J Baz B Wong HY Lee JS Khosrotehrani K. Accelerated endothelial to mesenchymal transition increased fibrosis *Via* deleting notch signaling in wound vasculature. J Invest Dermatol (2018) 138(5):1166–75. doi: 10.1016/j.jid.2017.12.004 29248546

[41] Link W. Introduction to foxo biology. *Methods Mol Biol* (2019) 1890:1–9. doi: 10.1007/978-1-4939-8900-3_1 30414140

[42] Liu W Li Y Luo B. Current perspective on the regulation of Foxo4 and its role in disease progression. Cell Mol Life Sci (2020) 77(4):651–63. doi: 10.1007/s00018-019-03297-w PMC11104957 31529218

[43] Zhou W Cao Q Peng Y Zhang QJ Castrillon DH DePinho RA. Foxo4 inhibits nf-kappab and protects mice against colonic injury and inflammation. Gastroenterology (2009) 137(4):1403–14. doi: 10.1053/j.gastro.2009.06.049 PMC2764529 19560465

[44] Zhao G Fu Y Cai Z Yu F Gong Z Dai R. Unspliced Xbp1 confers vsmc homeostasis and prevents aortic aneurysm formation *Via* Foxo4 interaction. Circ Res (2017) 121(12):1331–45. doi: 10.1161/CIRCRESAHA.117.311450 29089350

[45] Zhu M Zhang QJ Wang L Li H Liu ZP. Foxo4 inhibits atherosclerosis through its function in bone marrow derived cells. Atherosclerosis (2011) 219(2):492–8. doi: 10.1016/j.atherosclerosis.2011.09.038 PMC3226872 22005198

