# Supplementary material for: The role of FOXO4/NFAT2 signaling pathway in dysfunction of human coronary endothelial cells and inflammatory infiltration of vasculitis in Kawasaki disease
Source: Front Immunol. 2023 Jan 9;13:1090056. doi: 10.3389/fimmu.2022.1090056 (PMC9869249; doi:10.3389/fimmu.2022.1090056)

**The role of FOXO4/NFAT2 Signaling Pathway in Dysfunction of Human Coronary Endothelial Cells and Inflammatory Infiltration of Vasculitis in Kawasaki Disease**

**Short title:** FOXO4/NFAT2 signaling in Kawasaki Disease vasculitis

Hongbiao Huang1,2,6*, Jinfeng Dong3,*, Jiaqi Jiang1,*, Fang Yang2,*, Yiming Zheng1, Shuhui Wang1, Nana Wang1, Jin Ma1, Miao Hou1, Yueyue Ding1, Lijun Meng4, Wenyu Zhuo1, Daoping Yang1, Weiguo Qian1, Qiaobin Chen2, Guoping You5, Guanghui Qian1, Lei Gu6,# & Haitao Lv1,#

**Supplementary figures and tables**

**Supplementary Figures**

**Supplementary Figure 1**

**
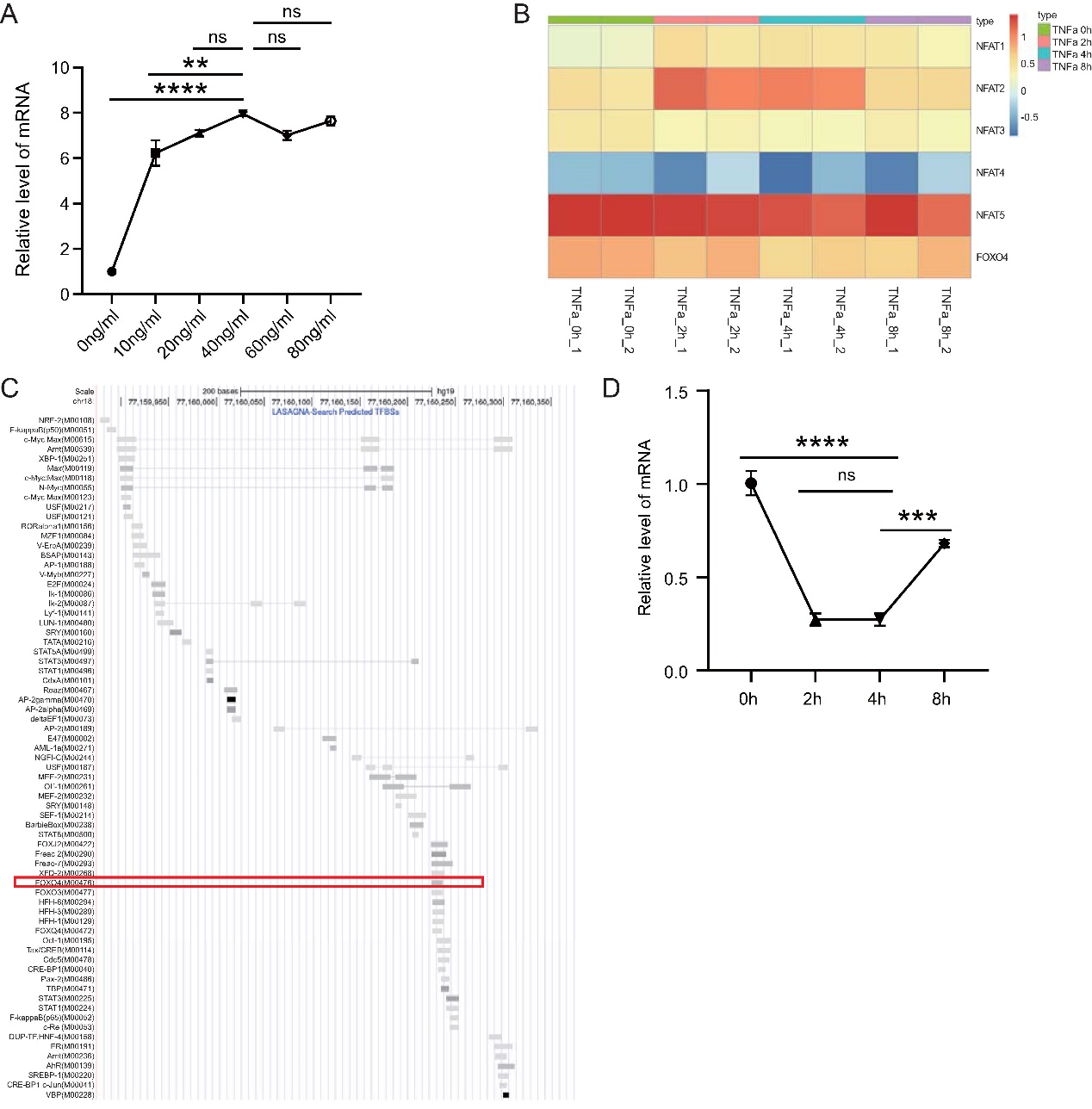
**

**Supplementary Figure 1.** FOXO4 interacts with NFAT2 in HCAECs. (A) qRT-PCR results of *NFAT2* in HCAECs stimulated by increase amounts of TNFα for 4 hours (n = 3). (B) Heatmap showing the relative expression levels of *FOXO4* and *NFAT* family members by the value of Log10 fragments per kilobase of transcript per million fragments mapped (FPKM) (n = 2). (C) Target genes predicted to interact with the promoter region of *NFAT2* according to the LASAGNA-Search 2.0 database. (D) qRT-PCR results for *FOXO4* in HCAECs stimulated by TNFα (40 ng/ml) at different timepoints (n = 3). Data are presented as the mean ± SEM. Quantitative data were analyzed using one-way ANOVA (A and D), *P < 0.05, **P < 0.01, ***P < 0.001, ****P < 0.0001

**Supplementary Figure 2.**


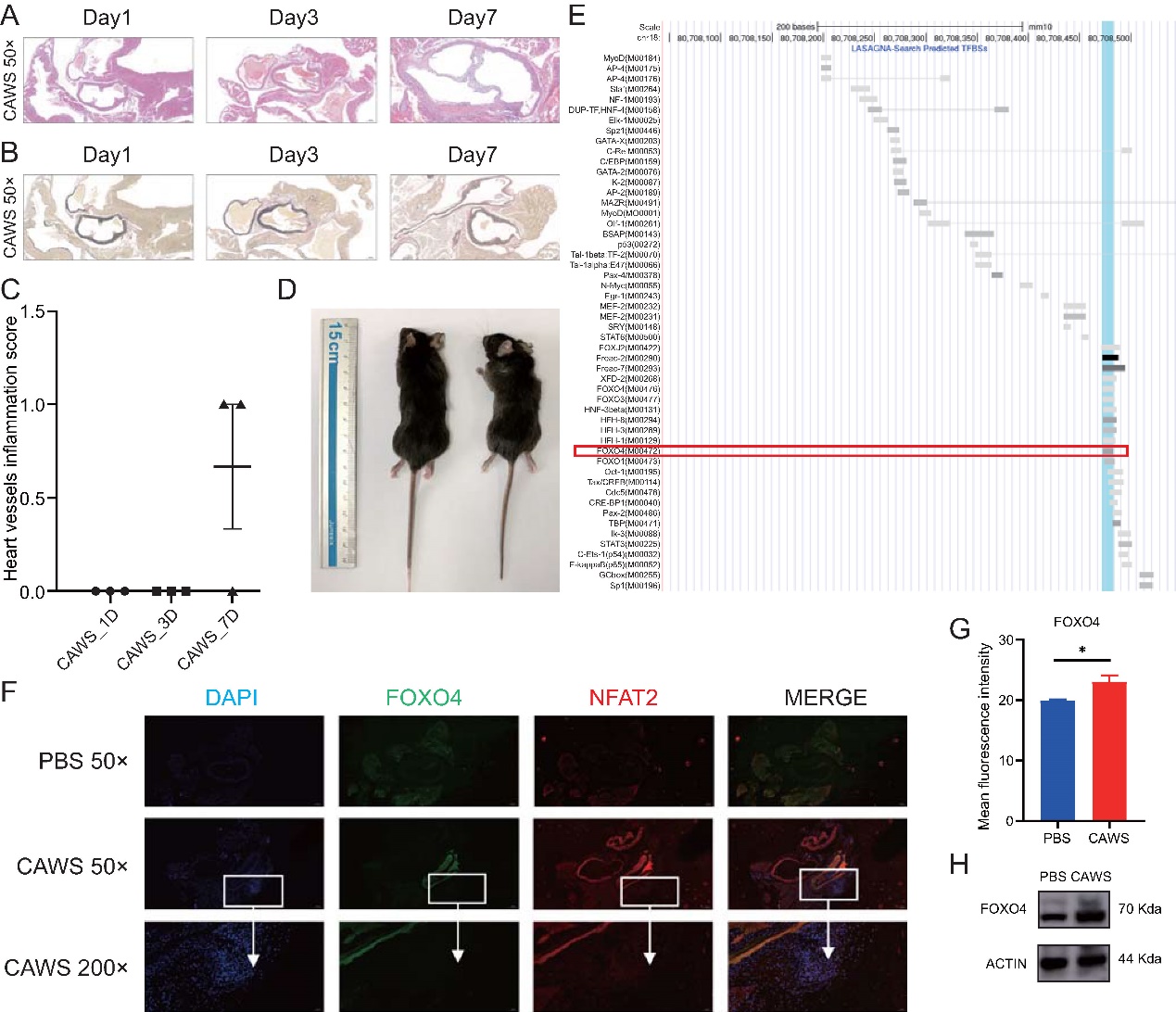


**Supplementary Figure 2.** Vasculitis and genes changes during the progression of KD in the CAWS-induced mouse model. At different timepoints the mice were sacrificed and heart tissues were harvested, cut into sections, and subjected to hematoxylin and eosin (HE)-staining (A) and elastic van Gieson (EVG)-staining (B). (C) heart vessel inflammation scores of WT mice injected with CAWS (n = 3). (D) At 14 days after PBS (left) and CAWS (right) injection, the mice were sacrificed. (E) Target mouse genes predicted to interact with the promoter region of *Nfat2* according to the LASAGNA-Search 2.0 database. (F) Immunofluorescent staining of FOXO4 (green) and NFAT2 (red) in heart sections from the PBS/CAWS-injected 28 days WT group mice. Scale bars, 200 µm (50×) and 10 µm (200×) in immunofluorescent staining. Nuclei were stained with DAPI in all images. (G) Quantification of FOXO4 in inflamed region (n = 3). (H) Western blotting assessment of FOXO4 levels in different groups. The loading control comprised β-Actin.

**Supplementary Figure 3**
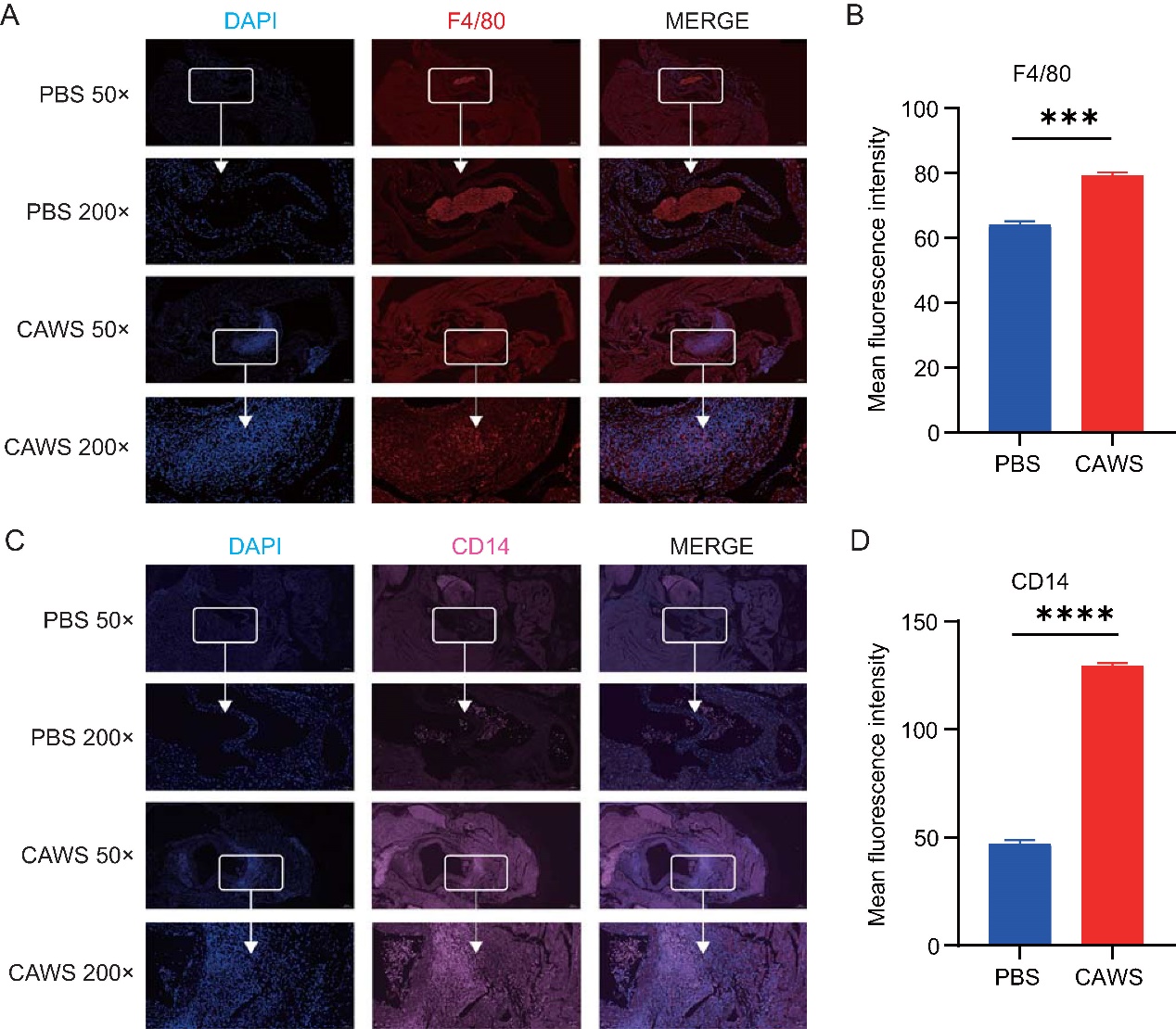


**Supplementary Figure 3.** Immune cell infiltration in the CAWS-induced mouse model. (A) Immunofluorescent staining of macrophage marker F4/80 (red) in heart sections from the PBS/CAWS-injected 14 days WT group mice. (B) Quantification of F4/80 in the inflamed region (n = 3). (C) Immunofluorescent staining of monocyte marker CD14 (pink) in heart sections from the PBS/CAWS-injected 14 days WT group mice. (D) Quantification of CD14 in the inflamed region (n = 3). Scale bars, 200 µm (50×) and 10 µm (200×) in immunofluorescent staining. Nuclei were stained with DAPI in all images.

**Supplementary Figure 4.**


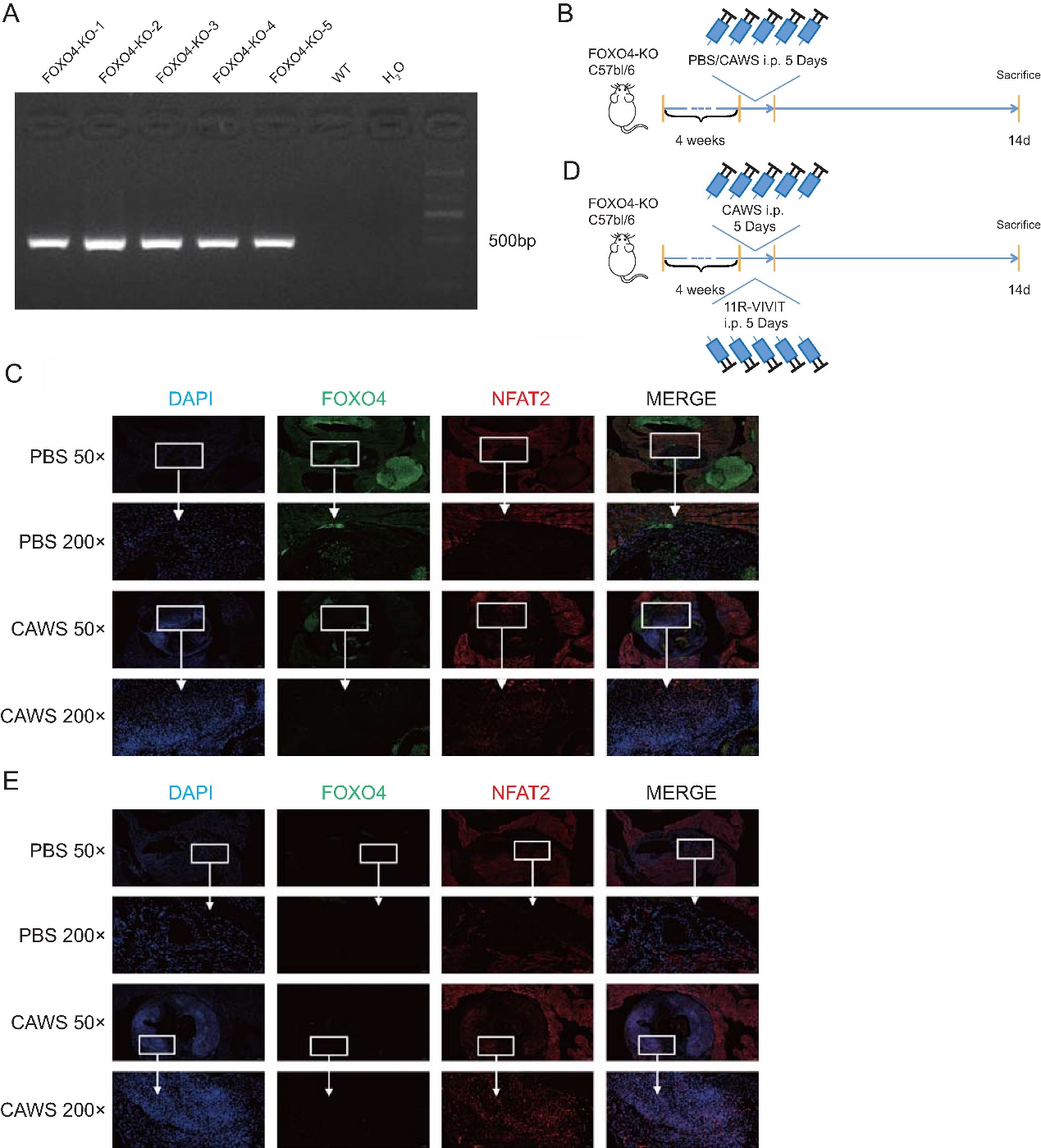


**Supplementary Figure 4.** NFAT2 expression levels in CAWS-induced FOXO4-KO mouse vasculitis. (A) DNA electrophoresis results of FOXO4-KO mice. (B) The protocol of the CAWS-induced FOXO4-KO mouse model was in this study. (C) Immunofluorescence staining for FOXO4 (green) and NFAT2 (red) in heart sections from PBS/CAWS-injected 14 days WT group mice. (D) The protocol for the CAWS plus 11R-VIVIT FOXO4-KO mouse model used in this research. (E) Immunofluorescent staining for FOXO4 (green) and NFAT2 (red) in heart sections from PBS and CAWS-injected 14 days FOXO4-KO group mice. Scale bars, 200 µm (50×) and 10 µm (200×) in immunofluorescent staining. Nuclei are stained with DAPI in all images.

**Supplementary Material and Tables**

**Primers**

**Human primers**

**NFAT1**

Forward Primer: GAGCCGAATGCACATAAGGTC

Reverse Primer: CCAGAGAGACTAGCAAGGGG

**NFAT2**

Forward Primer: CACCGCATCACAGGGAAGAC

Reverse Primer: GCACAGTCAATGACGGCTC

**NFAT3**

Forward Primer: GTCTTCCTTCCTCCTCCAGC

Reverse Primer: CTGAGTCCAGTTCTTCCCCC

**NFAT4**

Forward Primer: TATACCCGTTGAGTGCTCCC

Reverse Primer: CACTGAGGTCGTCCATCTTGT

**NFAT5**

Forward Primer: GGGTCAAACGACGAGATTGTG

Reverse Primer: GTCCGTGGTAAGCTGAGAAAG

**FOXO4**

Forward Primer: GGCTGCCGCGATCATAGAC

Reverse Primer: GGCTGGTTAGCGATCTCTGG

**NFAT2 promoter P1(-2931~-2820)**

Forward Primer: GAGTCTCTGGACATCGTGGG

Reverse Primer: AGCTTTTCCCATCAGGGGTC

**NFAT2 promoter P2(-2570~-2388)**

Forward Primer: CCTCCGAAGACGGGACG

Reverse Primer: TGTTCATCCACCCCGTCCA

**NFAT2 promoter P3(-2331~-2190)**

Forward Primer: AGCTTCTAGGGGTGTCTTTGC

Reverse Primer: CTAGGGGAGACGTTACACGG

**NFAT2 promoter P4(-2175~-2190)**

Forward Primer: AAACCTACGAGGAGGCAGGA

Reverse Primer: GGAATCCCGGGTCTGTTTCC

**NFAT2 promoter P5(-1263~-1049)**

Forward Primer: CGGGGTTCCATTTGTGCTGA

Reverse Primer: AGGCTAAACTCGTGTCTGCT

**NFAT2 promoter P6(-840~-697)**

Forward Primer: CAGTCGCGCCCATAAAACG

Reverse Primer: CCGGGACGTCGAGTTATTGT

**NFAT2 promoter P7(-41~+32)**

Forward Primer: TGTTTTCCAGCTTTAAAAAGGCA

Reverse Primer: GAGTTCGGAGCCTCTGAGT

**Mouse primers**

**NFAT1**

Forward Primer: TCATCCAACAACAGACTGCCC

Reverse Primer: GGGAGGGAGGTCCTGAAAACT

**NFAT2**

Forward Primer: GACCCGGAGTTCGACTTCG

Reverse Primer: TGACACTAGGGGACACATAACTG

**NFAT3**

Forward Primer: CACCACTTTGCTTACCACATCA

Reverse Primer: TTGGGACCACCTAATGGGCT

**NFAT4**

Forward Primer: GAGCTGGAATTTAAGCTGGTGT

Reverse Primer: CATGGAGGGGTATCCTCTGAG

**NFAT5**

Forward Primer: ATCGCCCAAGTCCCTGTACT

Reverse Primer: GCTTGTCTGACTCATTGATGCTA

**FOXO4**

Forward Primer: CTTCCTCGACCAGACCTCG

Reverse Primer: ACAGGATCGGTTCGGAGTGT

**FOXO4(for validate the effect of FOXO4-KO)**

Forward Primer: GAGAGAGTGCACCATAACCAATGA

Reverse Primer: GCAGGTGCTAGTAAACCTGATCC

**Statistical Analysis Data Tables**

**Supplemental Table I: Sample size and normality tests for data presented in the main figures.**

| Figure | Sample Group | Sample Size | Shapiro-Wilk test | Passed normality test |
| --- | --- | --- | --- | --- |
| Figure 1A | CON_NFAT1 | 15 | 0.2867 | Yes |
|  | CON_NFAT2 | 15 | 0.0053 | No |
|  | CON_NFAT3 | 15 | 0.1555 | Yes |
|  | CON_NFAT4 | 15 | 0.0136 | No |
|  | CON_NFAT5 | 15 | < 0.0001 | No |
|  | KD_NFAT1 | 15 | 0.0039 | No |
|  | KD_NFAT2 | 15 | 0.4591 | Yes |
|  | KD_NFAT3 | 15 | < 0.0001 | No |
|  | KD_NFAT4 | 15 | 0.0001 | No |
|  | KD_NFAT5 | 15 | 0.0002 | No |
| Figure 1C | 0h_NFAT1 | 3 | 0.1197 | Yes |
|  | 0h_NFAT2 | 3 | 0.7118 | Yes |
|  | 0h_NFAT3 | 3 | 0.8182 | Yes |
|  | 0h_NFAT4 | 3 | 0.5746 | Yes |
|  | 0h_NFAT5 | 3 | 0.8698 | Yes |
|  | 2h_NFAT1 | 3 | 0.0738 | Yes |
|  | 2h_NFAT2 | 3 | 0.1393 | Yes |
|  | 2h_NFAT3 | 3 | 0.6815 | Yes |
|  | 2h_NFAT4 | 3 | 0.4393 | Yes |
|  | 2h_NFAT5 | 3 | 0.1193 | Yes |
|  | 4h_NFAT1 | 3 | 0.8418 | Yes |
|  | 4h_NFAT2 | 3 | 0.3759 | Yes |
|  | 4h_NFAT3 | 3 | 0.4572 | Yes |
|  | 4h_NFAT4 | 3 | 0.2996 | Yes |
|  | 4h_NFAT5 | 3 | 0.3071 | Yes |
|  | 8h_NFAT1 | 3 | 0.3022 | Yes |
|  | 8h_NFAT2 | 3 | 0.7714 | Yes |
|  | 8h_NFAT3 | 3 | 0.0806 | Yes |
|  | 8h_NFAT4 | 3 | 0.9346 | Yes |
|  | 8h_NFAT5 | 3 | 0.8318 | Yes |
|  |  |  |  |  |
| Figure 2B | OE-CON | 3 | 0.6222 | Yes |
|  | OE-NFAT2 | 3 | 0.3577 | Yes |
| Figure 2D | KD-CON | 3 | 0.958 | Yes |
|  | sh1-NFAT2 | 3 | 0.4739 | Yes |
|  | sh2-NFAT2 | 3 | 0.9718 | Yes |
|  | sh3-NFAT2 | 3 | 0.2928 | Yes |
| Figure 2L | OE-CON | 3 | 0.9758 | Yes |
|  | OE-NFAT2 | 3 | 0.6811 | Yes |
|  | TNFα+OE-CON | 3 | 0.5123 | Yes |
|  | TNFα+OE-NFAT2 | 3 | 0.92 | Yes |
|  | KD-CON | 3 | 0.5199 | Yes |
|  | shNFAT2 | 3 | 0.9406 | Yes |
|  | TNFα+KD-CON | 3 | 0.9693 | Yes |
|  | TNFα+shNFAT2 | 3 | 0.6303 | Yes |
| Figure 2M | OE-CON-5d | 7 | 0.1219 | Yes |
|  | OE-NFAT2-5d | 7 | 0.1571 | Yes |
|  | KD-CON-5d | 7 | 0.5125 | Yes |
|  | shNFAT2-5d | 7 | 0.6437 | Yes |
|  | OE-CON-7d | 7 | 0.4448 | Yes |
|  | OE-NFAT2-7d | 7 | 0.9923 | Yes |
|  | KD-CON-7d | 7 | 0.233 | Yes |
|  | shNFAT2-7d | 7 | 0.1452 | Yes |
|  |  |  |  |  |
| Figure 3D | CON | 11 | 0.0076 | No |
|  | KD | 11 | 0.2453 | Yes |
| Figure 3F | CON | 3 | 0.3175 | Yes |
|  | FOXO4 0.2ug | 3 | 0.8791 | Yes |
|  | FOXO4 0.6ug | 3 | 0.7362 | Yes |
| Figure 3G | CON | 3 | 0.6228 | Yes |
|  | FOXO4 0.2ug | 3 | 0.5166 | Yes |
|  | FOXO4 0.6ug | 3 | 0.6271 | Yes |
| Figure 3J | CON | 3 | 0.0778 | Yes |
|  | FOXO4 0.2ug | 3 | 0.1796 | Yes |
|  | FOXO4 0.4ug | 3 | 0.4564 | Yes |
| Figure 3K | FOXO4_P3 | 5 | 0.1991 | Yes |
|  | IgG_P3 | 5 | N.S | No |
|  | FOXO4_P4 | 5 | 0.7454 | Yes |
|  | IgG_P4 | 5 | N.S | No |
|  | FOXO4_P5 | 5 | 0.0812 | Yes |
|  | IgG_P5 | 5 | N.S | No |
|  |  |  |  |  |
| Figure 4E | OE-CON | 3 | 0.9116 | Yes |
|  | OE-FOXO4 | 3 | 0.875 | Yes |
|  | TNFα+OE-CON | 3 | 0.8231 | Yes |
|  | TNFα+OE-FOXO4 | 3 | 0.5634 | Yes |
|  | KD-CON | 3 | 0.8829 | Yes |
|  | shFOXO4 | 3 | 0.8387 | Yes |
|  | TNFα+KD-CON | 3 | 0.697 | Yes |
|  | TNFα+shFOXO4 | 3 | 0.5434 | Yes |
| Figure 4F | OE-CON-5d | 7 | 0.8731 | Yes |
|  | OE-FOXO4-5d | 7 | 0.2988 | Yes |
|  | KD-CON-5d | 7 | 0.9493 | Yes |
|  | shFOXO4-5d | 7 | 0.1667 | Yes |
|  | OE-CON-7d | 7 | 0.807 | Yes |
|  | OE-FOXO4-7d | 7 | 0.6353 | Yes |
|  | KD-CON-7d | 7 | 0.1247 | Yes |
|  | shFOXO4-7d | 7 | 0.0857 | Yes |
|  |  |  |  |  |
| Figure 5B | PBS-Day 1-During Injection | 5 | 0.4009 | Yes |
|  | CAWS-Day 1-During Injection | 5 | 0.2866 | Yes |
|  | PBS-Day 2-During Injection | 5 | 0.4744 | Yes |
|  | CAWS-Day 2-During Injection | 5 | 0.5697 | Yes |
|  | PBS-Day 3-During Injection | 5 | 0.9318 | Yes |
|  | CAWS-Day 3-During Injection | 5 | 0.5853 | Yes |
|  | PBS-Day 4-During Injection | 5 | 0.9975 | Yes |
|  | CAWS-Day 4-During Injection | 5 | 0.3698 | Yes |
|  | PBS-Day 5-During Injection | 5 | 0.9105 | Yes |
|  | CAWS-Day 5-During Injection | 5 | 0.3139 | Yes |
|  | PBS-Day 1-After Injection | 5 | 0.2512 | Yes |
|  | CAWS-Day 1-After Injection | 5 | 0.0587 | Yes |
|  | PBS-Day 3-After Injection | 5 | 0.9982 | Yes |
|  | CAWS-Day 3-After Injection | 5 | 0.4313 | Yes |
|  | PBS-Day 7-After Injection | 5 | 0.6452 | Yes |
|  | CAWS-Day 7-After Injection | 5 | 0.7412 | Yes |
|  | PBS-Day 14-After Injection | 5 | 0.2126 | Yes |
|  | CAWS-Day 14-After Injection | 5 | 0.8421 | Yes |
|  | PBS-Day 28-After Injection | 5 | 0.1455 | Yes |
|  | CAWS-Day 28-After Injection | 5 | 0.9096 | Yes |
| Figure 5E | PBS | 5 | / | No |
|  | CAWS_14Day | 5 | 0.881 | Yes |
|  | CAWS_28Day | 5 | 0.9609 | Yes |
| Figure 5F | PBS_Day 1_NFAT2 | 3 | 0.9085 | Yes |
|  | CAWS_Day 1_NFAT2 | 3 | 0.7356 | Yes |
|  | PBS_Day 3_NFAT2 | 3 | 0.6439 | Yes |
|  | CAWS_Day 3_NFAT2 | 3 | 0.1613 | Yes |
|  | PBS_Day 7_NFAT2 | 3 | 0.8906 | Yes |
|  | CAWS_Day 7_NFAT2 | 3 | 0.6817 | Yes |
|  | PBS_Day 14_NFAT2 | 3 | 0.5732 | Yes |
|  | CAWS_Day 14_NFAT2 | 3 | 0.1335 | Yes |
|  | PBS_Day 28_NFAT2 | 3 | 0.2398 | Yes |
|  | CAWS_Day 28_NFAT2 | 3 | 0.8352 | Yes |
|  | PBS_Day 1_FOXO4 | 3 | 0.9126 | Yes |
|  | CAWS_Day 1_FOXO4 | 3 | 0.2101 | Yes |
|  | PBS_Day 3_FOXO4 | 3 | 0.4073 | Yes |
|  | CAWS_Day 3_FOXO4 | 3 | 0.2333 | Yes |
|  | PBS_Day 7_FOXO4 | 3 | 0.7069 | Yes |
|  | CAWS_Day 7_FOXO4 | 3 | 0.3654 | Yes |
|  | PBS_Day 14_FOXO4 | 3 | 0.3886 | Yes |
|  | CAWS_Day 14_FOXO4 | 3 | 0.1978 | Yes |
|  | PBS_Day 28_FOXO4 | 3 | 0.5974 | Yes |
|  | CAWS_Day 28_FOXO4 | 3 | 0.2132 | Yes |
| Figure 5J | FOXO4 (PBS) | 3 | 0.2873 | Yes |
|  | FOXO4 (CAWS) | 3 | 0.3734 | Yes |
|  | NFAT2 (PBS) | 3 | 0.5732 | Yes |
|  | NFAT2 (CAWS) | 3 | 0.1335 | Yes |
| Figure 5L | PBS-FOXO4 | 3 | 0.9631 | Yes |
|  | CAW-FOXO4 | 3 | 0.521 | Yes |
|  | PBS-NFAT2 | 3 | 0.9858 | Yes |
|  | CAWS-NFAT2 | 3 | 0.998 | Yes |
| Figure 5O | PBS | 3 | 0.766 | Yes |
|  | CAWS | 3 | 0.3768 | Yes |
|  |  |  |  |  |
| Figure 6A | NFAT1(CAWS) | 6 | 0.9956 | Yes |
|  | NFAT1(CAWS+11R) | 6 | 0.9566 | Yes |
|  | NFAT2(CAWS) | 6 | 0.3477 | Yes |
|  | NFAT2(CAWS+11R) | 6 | 0.1108 | Yes |
|  | NFAT3(CAWS) | 6 | 0.574 | Yes |
|  | NFAT3(CAWS+11R) | 6 | 0.7413 | Yes |
|  | NFAT4(CAWS) | 6 | 0.5143 | Yes |
|  | NFAT4(CAWS+11R) | 6 | 0.0429 | No |
|  | NFAT5(CAWS) | 6 | 0.0447 | No |
|  | NFAT5(CAWS+11R) | 6 | 0.0693 | Yes |
| Figure 6F | CAWS | 4 | 0.2725 | Yes |
|  | CAWS+DMSO | 4 | 0.0239 | No |
|  | CAWS+11R | 4 | 0.85 | Yes |
| Figure 6I | PBS | 4 | 0.8573 | Yes |
|  | CAWS | 4 | 0.8395 | Yes |
|  | CAWS+DMSO | 4 | 0.1248 | Yes |
|  | CAWS+11R | 4 | 0.9238 | Yes |
|  |  |  |  |  |
| Figure 7D | CAWS | 5 | > 0.1000 | Yes |
|  | FOXO4-KO+CAWS | 5 | > 0.1000 | Yes |
| Figure 7E | PBS | 5 | > 0.1000 | Yes |
|  | CAWS | 5 | > 0.1000 | Yes |
|  | FOXO4-KO+PBS | 5 | 0.0565 | Yes |
|  | FOXO4-KO+CAWS | 5 | > 0.1000 | Yes |
| Figure 7K | FOXO4-KO+CAWS | 5 | > 0.1000 | Yes |
|  | FOXO4-KO+CAWS+11R | 5 | > 0.1000 | Yes |
| Figure 7M | FOXO4-KO+PBS | 5 | > 0.1000 | Yes |
|  | FOXO4-KO+CAWS | 5 | > 0.1000 | Yes |
|  | FOXO4-KO+CAWS+11R | 5 | > 0.1000 | Yes |

**Table II: Statistical tests and P values for the data presented in the main figures.**

| Figure | Statistical Test | Sample | P value |
| --- | --- | --- | --- |
| Figure 1A | Kruskal-Wallis test with Dunn’s multiple comparisons test | NFAT1 *vs*. NFAT2 | 0.0018 |
|  |  | NFAT2 *vs*. NFAT3 | < 0.0001 |
|  |  | NFAT2 *vs*. NFAT4 | < 0.0001 |
|  |  | NFAT2 *vs*. NFAT5 | 0.0361 |
| Figure 1D | One-way Anova with Tukey's multiple comparisons test | 0h_NFAT1 *vs*. 0h_NFAT2 | >0.9999 |
|  |  | 0h_NFAT2 *vs*. 0h_NFAT3 | >0.9999 |
|  |  | 0h_NFAT2 *vs*. 0h_NFAT4 | >0.9999 |
|  |  | 0h_NFAT2 *vs*. 0h_NFAT5 | >0.9999 |
|  |  | 2h_NFAT1 *vs*. 2h_NFAT2 | < 0.0001 |
|  |  | 2h_NFAT2 *vs*. 2h_NFAT3 | < 0.0001 |
|  |  | 2h_NFAT2 *vs*. 2h_NFAT4 | < 0.0001 |
|  |  | 2h_NFAT2 *vs*. 2h_NFAT5 | < 0.0001 |
|  |  | 4h_NFAT1 *vs*. 4h_NFAT2 | < 0.0001 |
|  |  | 4h_NFAT2 *vs*. 4h_NFAT3 | < 0.0001 |
|  |  | 4h_NFAT2 *vs*. 4h_NFAT4 | < 0.0001 |
|  |  | 4h_NFAT2 *vs*. 4h_NFAT5 | < 0.0001 |
|  |  | 8h_NFAT1 *vs*. 8h_NFAT2 | < 0.0001 |
|  |  | 8h_NFAT2 *vs*. 8h_NFAT3 | < 0.0001 |
|  |  | 8h_NFAT2 *vs*. 8h_NFAT4 | < 0.0001 |
|  |  | 8h_NFAT2 *vs*. 8h_NFAT5 | < 0.0001 |
|  |  |  |  |
| Figure 2B | Unpaired t test (two-tailed) | OE-NFAT2 *vs*. OE-CON | < 0.0001 |
| Figure 2D | Unpaired t test (two-tailed) | sh1-NFAT2 *vs*. KD-CON | 0.0003 |
|  |  | sh2-NFAT2 *vs*. KD-CON | 0.0001 |
|  |  | sh3-NFAT2 *vs*. KD-CON | 0.0005 |
| Figure 2L | One-way ANOVA with Tukey's multiple comparisons test | OE-CON *vs*. OE-NFAT2 | 0.0011 |
|  |  | TNFα+OE-CON *vs*. TNFα+OE-NFAT2 | 0.8823 |
|  |  | OE-CON *vs*. TNFα+OE-CON | < 0.0001 |
|  |  | OE-NFAT2 *vs*. TNFα+OE-NFAT2 | < 0.0001 |
|  |  | KD-CON *vs*. shNFAT2 | 0.4925 |
|  |  | TNFα+KD-CON *vs*. TNFα+shNFAT2 | 0.001 |
|  |  | KD-CON *vs*. TNFα+KD-CON | < 0.0001 |
|  |  | shNFAT2 *vs*. TNFα+shNFAT2 | 0.0056 |
| Figure 2M | One-way ANOVA with Tukey's multiple comparisons test | OE-CON-5d *vs*. OE-NFAT2-5d | < 0.0001 |
|  |  | KD-CON-5d *vs*. shNFAT2-5d | 0.7793 |
|  |  | OE-CON-7d *vs*. OE-NFAT2-7d | 0.0002 |
|  |  | KD-CON-7d *vs*. shNFAT2-7d | 0.0114 |
|  |  |  |  |
| Figure 3D | Mann-Whitney test (two-tailed) | CON *vs*. KD | 0.0004 |
| Figure 3F | One-way ANOVA with Tukey's multiple comparisons test | CON *vs*. FOXO4 0.2 μg | < 0.0001 |
|  |  | CON *vs*. FOXO4 0.6 μg | < 0.0001 |
|  |  | FOXO4 0.2ug *vs*. FOXO4 0.6 μg | 0.1464 |
| Figure 3G | One-way Anova with Tukey's multiple comparisons test | CON *vs*. FOXO4 0.2 μg | 0.0503 |
|  |  | CON *vs*. FOXO4 0.6 μg | 0.0005 |
|  |  | FOXO4 0.2ug *vs*. FOXO4 0.6 μg | 0.0062 |
| Figure 3J | One-way ANOVA with Tukey's multiple comparisons test | CON *vs*. FOXO4 0.2 μg | 0.0018 |
|  |  | CON *vs*. FOXO4 0.4 μg | 0.0007 |
|  |  | FOXO4 0.2 μg *vs*. FOXO4 0.4 μg | 0.5215 |
| Figure 3K | Mann-Whitney test (two-tailed) | FOXO4_P3 vs. IgG_P3 | 0.0079 |
|  |  | FOXO4_P4 vs. IgG_P4 | 0.0079 |
|  |  | FOXO4_P5 vs. IgG_P5 | 0.0079 |
|  |  |  |  |
| Figure 4E | One-way ANOVA with Tukey's multiple comparisons test | OE-CON *vs*. OE-FOXO4 | 0.0002 |
|  |  | TNFα+OE-CON *vs*. TNFα+OE-FOXO4 | 0.1602 |
|  |  | OE-CON *vs*. TNFα+OE-CON | < 0.0001 |
|  |  | OE-FOXO4 *vs*. TNFα+OE-FOXO4 | < 0.0001 |
|  |  | KD-CON *vs*. shFOXO4 | < 0.0001 |
|  |  | TNFα+KD-CON *vs*. TNFα+shFOXO4 | 0.9888 |
|  |  | KD-CON *vs*. TNFα+KD-CON | < 0.0001 |
|  |  | shFOXO4 *vs*. TNFα+shFOXO4 | 0.0019 |
| Figure 4F | One-way ANOVA with Tukey's multiple comparisons test | OE-CON-5d *vs*. OE-FOXO4-5d | 0.0849 |
|  |  | KD-CON-5d *vs*. shFOXO4-5d | 0.8611 |
|  |  | OE-CON-7d *vs*. OE-FOXO4-7d | < 0.0001 |
|  |  | KD-CON-7d *vs*. shFOXO4-7d | 0.0067 |
|  |  |  |  |
| Figure 5B | Unpaired t test (two-tailed) | PBS-Day 1-During Injection *vs*. CAWS-Day 1-During Injection | 0.5463 |
|  |  | PBS-Day 2-During Injection *vs*. CAWS-Day 2-During Injection | 0.3606 |
|  |  | PBS-Day 3-During Injection *vs*. CAWS-Day 3-During Injection | 0.1883 |
|  |  | PBS-Day 4-During Injection *vs*. CAWS-Day 4-During Injection | 0.5157 |
|  |  | PBS-Day 5-During Injection *vs*. CAWS-Day 5-During Injection | 0.7267 |
|  |  | PBS-Day 1-After Injection *vs*. CAWS-Day 1-After Injection | 0.8042 |
|  |  | PBS-Day 3-After Injection *vs*. CAWS-Day 3-After Injection | 0.3601 |
|  |  | PBS-Day 7-After Injection *vs*. CAWS-Day 7-After Injection | 0.157 |
|  |  | PBS-Day 14-After Injection *vs*. CAWS-Day 14-After Injection | 0.0018 |
|  |  | PBS-Day 28-After Injection *vs*. CAWS-Day 28-After Injection | 0.6436 |
| Figure5E | Mann-Whitney test (two-tailed) | PBS vs. CAWS_14Day | 0.0079 |
|  | Unpaired t test (two-tailed) | CAWS_14Day vs. CAWS_28Day | 0.0007 |
| Figure5F | Unpaired t test (two-tailed) | PBS_Day1_NFAT2 vs. CAWS_Day1_NFAT2 | 0.0657 |
|  |  | PBS_Day3_NFAT2 vs. CAWS_Day3_NFAT2 | 0.4595 |
|  |  | PBS_Day7_NFAT2 vs. CAWS_Day7_NFAT2 | 0.0034 |
|  |  | PBS_Day14_NFAT2 vs. CAWS_Day14_NFAT2 | 0.0023 |
|  |  | PBS_Day28_NFAT2 vs. CAWS_Day28_NFAT2 | 0.0005 |
| Figure5G | Unpaired t test (two-tailed) | PBS_Day1_FOXO4 vs. CAWS_Day1_FOXO4 | 0.0246 |
|  |  | PBS_Day3_FOXO4 vs. CAWS_Day3_FOXO4 | 0.0077 |
|  |  | PBS_Day7_FOXO4 vs. CAWS_Day7_FOXO4 | 0.0002 |
|  |  | PBS_Day14_FOXO4 vs. CAWS_Day14_FOXO4 | 0.0035 |
|  |  | PBS_Day28_FOXO4 vs. CAWS_Day28_FOXO4 | 0.0031 |
| Figure5J | Unpaired t test (two-tailed) | FOXO4(CAWS) vs. FOXO4(PBS) | 0.0176 |
|  |  | NFAT2(CAWS) vs. NFAT2(PBS) | 0.0023 |
| Figure5L | Unpaired t test (two-tailed) | FOXO4(CAWS) vs. FOXO4(PBS) | <0.0001 |
|  |  | NFAT2(CAWS) vs. NFAT2(PBS) | <0.0001 |
| Figure5O | Unpaired t test (two-tailed) | CAWS vs. PBS | 0.0064 |
| Figure6A | Unpaired t test (two-tailed) | NFAT1 CAWS vs. CAWS+11R | 0.0888 |
|  | Unpaired t test (two-tailed) | NFAT2 CAWS vs. CAWS+11R | 0.0016 |
|  | Unpaired t test (two-tailed) | NFAT3 CAWS vs. CAWS+11R | 0.5704 |
|  | Mann-Whitney test (two-tailed) | NFAT4 CAWS vs. CAWS+11R | 0.4199 |
|  | Mann-Whitney test (two-tailed) | NFAT5 CAWS vs. CAWS+11R | 0.2879 |
| Figure6F | Kruskal-Wallis test with Dunn’s multiple comparisons test | CAWS vs. CAWS+DMSO | >0.9999 |
|  |  | CAWS vs. CAWS+11R | 0.1389 |
|  |  | CAWS+DMSO vs. CAWS+11R | 0.0158 |
| Figure6I | One-way Anova with Tukey's multiple comparisons test | PBS vs. CAWS | 0.0025 |
|  |  | PBS vs. CAWS+DMSO | 0.0003 |
|  |  | PBS vs. CAWS+11R | 0.0632 |
|  |  | CAWS vs. CAWS+DMSO | 0.5925 |
|  |  | CAWS+DMSO vs. CAWS+11R | 0.0363 |
| Figure7D | Unpaired t test (two-tailed) | CAWS vs. FOXO4-KO+CAWS | 0.0028 |
| Figure7E | One-way Anova with Tukey's multiple comparisons test | PBS vs. CAWS | 0.031 |
|  |  | PBS vs. FOXO4-KO+PBS | >0.9999 |
|  |  | CAWS vs. FOXO4-KO+CAWS | 0.5299 |
|  |  | FOXO4-KO+PBS vs. FOXO4-KO+CAWS | 0.002 |
| Figure7K | Unpaired t test (two-tailed) | FOXO4-KO+CAWS vs. FOXO4-KO+CAWS+11R | 0.0048 |
| Figure7M | One-way Anova with Tukey's multiple comparisons test | FOXO4-KO+PBS vs. FOXO4-KO+CAWS | 0.0026 |
|  |  | FOXO4-KO+PBS vs. FOXO4-KO+CAWS+11R | 0.4721 |
|  |  | FOXO4-KO+CAWS vs. FOXO4-KO+CAWS+11R | 0.0223 |

**Supplementary Methods**

*1.1* Sampling human blood

Blood was sampled from healthy controls, patients with fever (such as pneumonia, bronchitis, or gastroenteritis), and patients with KD at the Soochow University Affiliated Children's Hospital. All blood samples were collected before initial treatment. The study was carried out following the tenets of the Declaration of Helsinki and the Ethics Committee of Soochow University Affiliated Children's Hospital approved the study (Suzhou, China; approval no. 2020CS075). The Ethics Committee informed all the participants and their parents about the study details, who then provided written informed consent.

*1.2 Genetically engineered mice*

The animal experiments were carried out according to the National Institutes of Health Guide for the Care and Use of Laboratory Animals. The Animal Care and Use Committees of Soochow University approved the animal studies. *Foxo4* knockout mice (C57BL/6J) were constructed using CRISPR/Cas-mediated genome engineering and were provided by Cyagen Biosciences (Guangzhou, China). The *Foxo4* gene (transcript: (ENSMUST00000062000) has three exons: exon 1 contains the ATG start codon and exon 3 has the TGA stop codon. Exon 1–3 were selected as the target sites. Cas9 and gRNA were co-injected into fertilized eggs to produce for *Foxo4* knockout (KO) mice.

*1.3 Preparation of CAWS*

The CAWS was prepared from *Candida albicans* strain NBRC1385 using previously described methods (1-3). *C. albicans* was grown in C-limiting medium for 48 hours at 26 °C with rotation at 250 rpm. Thereafter, we added an equal volume of ethanol and the culture was stored in a 4 °C refrigerator at overnight. The cultures were then centrifuged and the pellet was resuspended in water with stirring for 2 h. The solution subjected to repeated centrifugation, the supernatant was retained, added with an equal volume of ethanol, and left at 4 °C overnight. After further centrifugation, the precipitate was dried under acetone for 2 days. The resultant CAWS was dissolved in phosphate-buffered saline (PBS) before use.

*1.4 CAWS-induced vasculitis in mouse model*

For the KD animal model, JOINN Laboratories (Suzhou, China) provided C57BL/6 mice (male, 3–4 weeks old). The mice were housed in standard experimental cages and reared under controlled conditions (temperature = 25 ± 2 °C; humidity = 50 ± 5%). The mice were divided randomly into eight groups (n = 3–5 per group): A PBS group, a CAWS group, a CAWS + 11R-VIVIT group, a CAWS + Dimethyl sulfoxide (DMSO, Merck, Darmstadt, Germany) group, a FOXO4-KO + PBS group, a FOXO4-KO + CAWS group, a FOXO4-KO + CAWS + 11R-VIVIT group, and a FOXO4-KO + CAWS + DMSO group. In the groups receiving 11R-VIVIT pretreatment, we injected 11R-VIVIT intraperitoneally 1 h before CAWS (4 mg/head). The treatments were repeated on 5 consecutive days. At 1–28 days after the last CAWS injection, the mice were anesthetized by inhaling 1.5–2% isoflurane and sacrificed to harvest their PBMCs and hearts. All animal experiments were carried out following the Guide for the Care and Use of Laboratory Animals of the China National Institutes of Health, and the Animal Care and Use Committee of Soochow University approved the experiments (approval number: SUDA20220906A01).

*1.5 Histology and* *Immunohistochemical staining*

Mouse hearts were dissected out fixed using 4% paraformaldehyde, embedded in paraffin, and cut into 0.5 μm thick sections. The sections were stained using hematoxylin and eosin (HE) and elastic van Gieson (EVG) staining as described previously (4). A mouse monoclonal antibody against Cadherin 5 (CDH5) (66804-1-Ig; Proteintech, Wuhan, China) was reacted with the sections. After further processing, the sections were imaged under a microscope (Nikon DS-Ri2, Tokyo, Japan). The severity of inflammatory infiltration was evaluated using heart vessel inflammation scores (5). CDH5 quantification for each sample was determined by a pathologist blinded to sample information using modified H-scores ([{% of weak staining} * 1] + [{% of moderate staining}*2] + [{% of strong staining}*3]), which determined the overall percentage of CHD5 positivity across the perivascular region, yielding a range from 0 to 300 (6).

*1.6 Immunofluorescence staining*

Mouse heart samples were incubated with primary rabbit anti-FOXO4 (21535-1-AP), mouse anti-NFAT2 (66963-1-Ig) antibodies, rabbit anti-CD14 (17000-1-AP) (all Proteintech) and rabbit anti-F4/80 (70076) (Cell Signaling Technology, Inc., Danvers, MA, USA)). Cell samples were incubated with primary rabbit anti-CDH5 (ab33168; Abcam, Cambridge, MA, USA). The secondary antibodies comprised Alexa Fluor 488 goat anti-rabbit/mouse (GB25303/GB21301; Servicebio, Wuhan, China) A laser scanning confocal microscope (Olympus FV1200, Tokyo, Japan) was used to image the samples. Nuclei were stained using 4′,6-diamidino-2-phenylindole (DAPI). The relative abundance of CDH5 in cell samples was estimated using the immunofluorescence area divided by the number of cells. The immunofluorescence area was calculated using ImageJ (NIH, Bethesda, MD, USA).

*1.7 Cell culture*

HCAECs were grown in Roswell Park Memorial Institute (RPMI)‑1640 medium (Biosharp Corp., Seoul, South Korea) and human embryonic kidney 293T (HEK293T) cells were grown in Dulbecco’s modified Eagle’s medium (DMEM)/High glucose medium (Biosharp Corp.). Both media were added with 10% fetal bovine serum (FBS; Biological Industries, Kibbutz Beit Haemek, Israel) and 100 U/ml penicillin‑G and 100 mg/ml streptomycin. The cells were cultured in 5% CO_2_ and 95% humidified air at 37 °C. The Cell Bank of Type Culture Collection of The Chinese Academy of Sciences provided both cell lines.

*1.8 FOXO4 knockdown, FOXO4 overexpression, NFAT2 knockdown and NFAT2 overexpression in HCAEC cells*

IGE Biotechnology, Ltd. (Guangzhou, China) designed and produced the *FOXO4* (NM_005938.4) overexpression and short hairpin RNA (shRNA) lentiviruses, based on vectors PLVX and PLKO.1, respectively. To prepare the lentiviruses, we purchased the envelop and packaging plasmids (pMD2.G, 12259; psPAX2, 12260) from Addgene, Inc. (Watertown, MA, USA). We packaged the lentiviruses according to a previously described method (7). 293T cells were transfected when they reached approximately 70% confluence. We mixed the expression and packaging plasmids at a ratio of 1:1. Then, we added 15 μl of 1 mg/ml polyethylenimine (Proteintech) to the plasmids, mixed them, and incubated them for 20 min at room temperature. Subsequently, we added the transfection mixture to a 10 cm cell culture dish containing 70% confluent 293T cells, followed by incubation at 37 °C. At 6 hours after transfection, we replaced the transfection complex-containing medium with fresh culture medium. Forty-eight hours later, the lentiviruses were harvested. The prepared lentiviruses were transfected into HCAECs with selection using 0.8 μg/ml puromycin (Invitrogen; Thermo Fisher Scientific, Inc., Waltham, MA, USA) for 7–10 days. We purchased the *NFAT2* (NM_006162) overexpression, knockdown, and control lentiviruses from GeneChem Corporation (Shanghai, China). Cell transfection (with puromycin selection) was carried out following the supplier's protocol. All operations using lentiviruses were carried out in a biological safety cabinet, and complied with the operating safety standards for the use of lentiviruses.

*1.9 Stimulation of cultured HCAECs with tumor necrosis factor-α (TNF-α)*

At 5 ×10^4^ cells per well, HCAECs were seeded the wells of a 12‑well culture plate and cultured for 18 h in 5% CO_2_ and 95% humidified air at 37 °C. The HCAECs were then challenged with 0~80 ng/ml TNFα (Ag24020; Proteintech) for 0~8 hours. Then, the most appropriate time and dose of TNFα was selected according to the expression level of NFAT2.

*1.10 RNA extraction and quantitative real-time reverse transcription PCR (qRT-PCR)*

The TRIzol® reagent (Thermo Fisher Scientific Inc.) was employed to extract total RNA. Subsequently, PrimeScript RT Master mix (Takara Bio, Inc., Dalian, China) was used to reverse transcribe the mRNA to cDNA at 37 °C for 15 min followed by 85 °C for 10 sec. The cDNA was diluted four-fold RNase‑free water. Next, the cDNA was quantified using a LightCycler 480 II Real‑Time PCR system (Roche Diagnostics, Basel, Switzerland) and a SYBR Green qPCR Master mix (Roche Diagnostics) at 50°C for 2 min, followed by the thermal cycling profile of 95 °C for 10 min; 50 cycles of 95 °C for 20 s, 65 °C for 20 s, and 72°C for 20 s. The mouse and human mRNAs encoding glyceraldehyde-3-phosphate dehydrogenase (GAPDH) were quantified as internal controls. The 2‑ΔΔCt method (8) was used to determine the relative expression of target genes in different samples. The primers for the human and mouse genes are shown in the Supporting Information.

*1.11 Western blotting*

HCAEC cells and heart tissues were homogenized in Radioimmunoprecipitation assay (RIPA) lysis buffer (Beyotime Institute of Biotechnology, Jiangsu, China) with phosphatase and protease inhibitors (Roche Diagnostics) followed by 5 min of ultrasonication on ice. The samples were centrifuged at 13800× *g* and 4 °C for 15 min, and the proteins in the supernatant were quantified using a bicinchoninic acid (BCA) assay kit (Thermo Fisher Scientific, Inc.). 8% gradient sodium dodecyl sulfate polyacrylamide gel electrophoresis (SDS-PAGE; Genscript, Piscataway, NJ, USA) was used to resolve equal amounts of protein, which were then electrotransferred onto a polyvinylidene fluoride (PVDF) membrane (MilliporeSigma, Burlington, M, USA). Skimmed milk (5% in 0.1% TBS-Tween 20 (TBST)) was used to block the membranes at room temperature for 2 h, followed by incubation with primary antibodies overnight at 4 °C. Subsequently, three washes with TBST were carried out, followed by incubation of the membranes with goat anti-rabbit (111-035-003), anti‑mouse IgG (H+L; 115035-003) secondary antibodies conjugated with horseradish peroxidase (HRP) (both 1:5,000; both Jackson ImmunoResearch Laboratories, Inc., West Grove, PA, USA) and HRP-Donkey Anti-Goat IgG secondary antibodies (H+L; SA00001-3; Proteintech) for 1 h at room temperature. The immunoreactive protein bands were visualized using an Amersham Imager 600 (GE Healthcare Life Sciences, Little Chalfont, UK) and quantified using ImageJ. The following primary antibodies were used: anti-FOXO4 (9472S), anti-NFAT2 (8032S), anti-β-actin (3700S), anti‑FLAG (AS153036), (all 1:1,000; all Cell Signaling Technology, Inc.), anti-CDH5 (AF1002; R&D Technology, Inc., North Kingstown, RI, USA), and anti-GAPDH (AP0063; 1:5,000; Bioworld Technology Inc., St Louis Park, MN, USA).

*1.12 Luciferase Assay*

HEK293T cells were added to 24-well plates and subjected to co-transfection using the indicated protein coding plasmids and NFAT luciferase reporter plasmids (11512ES03, TEASEN Biotechnology Ltd., Shanghai, China) driven by the NFAT gene fragments. NFAT2_Lucferase (NM_005938) was designed according to the results of ChIP-Seq, Renilla luciferase expressing plasmids were co-transfected and used for normalization as internal controls. The total amount of the plasmids was maintained by adding an equal dose of the corresponding empty vectors. JetPRIME (PT-114-15, Polyplus Transfection) was used to transfect the plasmids for 6 h. HEK293T cells were maintained normally for 72 h after plasmid transfection. Then, the transfected cells were washed three times and harvested in passive lysis buffer (E1940, Promega, Madison, WI, USA) Finally, a dual luciferase assay kit (E1940, Promega) was used to assay both firefly and renilla luciferase activities in the supernatants following the supplier's protocol. The Stop & Glo® Reagent (Promega) was used to detect fluorescent light emission. Two replicates of each sample were tested to exclude variations. The results are shown as the firefly/renilla luciferase activity ratio and represent at least two independent experiments.

*1.13 Chromatin Immunoprecipitation (ChIP) Assay*

The ChIP assay was carried out according to a previously described method (9). HCAECs overexpressing *FOXO4* were seeded in five 10-cm plates. After washing with PBS twice, 1% formaldehyde was used to fix the cells at room temperature for 10 min at room with gentle shaking. After centrifugation for 5 min at 200 × *g* , the pelleted cells were lysed in ice cold cell lysis buffer (0.01 mM NaCl, 0.5 M EDTA pH 7.5, 1 M Tris, pH 7.5, 0.2% NP-40) with a protease inhibitor cocktail (Sigma, Darmstadt, Germany). Thereafter, cells rupture was induced by gentle aspiration of the cells through a 1 ml insulin needle twice. After centrifugation for 5 min at 13,800 × *g*, the pellet was suspended in shearing buffer (20% SDS, 0.5 M EDTA pH 8.0, 1 M Tris pH 8.0) with a protease inhibitor cocktail, and then an ultrasonic cell disruptor (M220, Covaris, Woburn, MA, USA) delivered sonication for 5 minutes and 30 seconds at 4 °C, which sheared the genomic DNA into fragments of 500–1000 bp. After centrifugation for 5 min at 13,800 × *g* and 4 °C, the supernatants were separated into two parts, one part was pre-incubated with an anti-FOXO4 antibody (720154, Regeneron, Tarrytown, NY, USA) and the other part was incubated with IgG (A7028, Beyotime) overnight at 4 °C with gentle shaking. Subsequently, we added Dynabeads (Protein G beads) (10004D, Thermo Fisher Scientific) to the samples to immunoprecipitate the complexes for 4 h at 4 °C. The antibody-chromatin complexes on the beads were washed using lysis buffer six times, and with TE buffer (Sigma) twice. Next, EB buffer (1 M NaHCO_3_, 20% SDS) was used to disassociate the antibody-chromatin complexes from beads, followed by reverse crosslinking in 5 M NaCl overnight at 65 °C. Contaminating RNA was removed by incubation with 10 mg/ml RNase (#7013, Cell Signaling Technology)) for 30 min at 37 °C. Proteins were removed by incubation for 1 h at 45 °C in 20 mg/ml Proteinase K (AM2546, Invitrogen), 0.5 M EDTA (pH 8.0) and 1 M Tris HCl (pH 8.0). A PCR Purification Kit (250) (QIAGEN, Hilden, Germany) was used to purify the DNA fragments, followed by gel electrophoresis to visualize the size of DNA fragments. In the ChIP‑qPCR analyses, the values from the immunoprecipitated (IP) samples were normalized to that from the IgG-DNA reaction. Primer sequences are available on request.

*1.14 RNA sequencing (RNA-seq)*

Total RNAs from the HCAEC cells of the control, the TNFα-stimulated groups, and the overexpression and knockdown groups were isolated using TRIzol (Invitrogen). The amount and integrity of the total RNA were assessed using an RNA 6000 Nano Kit in a Bioanalyzer 2100 system (Agilent Technologies, Santa Clara, CA, USA). Poly T oligo-attached magnetic beads (Beckman Coulter, Beverly, CA, USA) were then used to purify the mRNA. The RNA-seq libraries were prepared using a NEBNext® Ultra™ RNA Library Prep Kit for Illumina® (Illumina, San Diego, CA, USA), and sequenced using the Illumina NovaSeq 6000 system and the data filtering was carried out by Novogene Bioinformatics Technology Co., Ltd (Beijing, China).

*1.15* *Cell proliferation assays*

Nine groups of HCAEC cells were subjected to cell proliferation assays, including the FOXO4/NFAT2 overexpression and knockdown groups, their respective controls, and WT HCAECs as normal controls. All lentivirus-infected HCAECs were tested 2 weeks after transfection. The different HCAEC groups were added to the wells of a 96‑well plate at 5 × 10^3^ cells per well and incubated in 5% CO_2_ and 95% humidified air at 37 °C for 24 and 48 h. Subsequently, cell viability was determined using a Cell Counting Kit-8 (CCK-8) assay (Dojindo Molecular Technologies, Inc., Kumamoto, Japan), in which a scanning multi-well spectrophotometer (Bio-Rad Model 550; Bio‑Rad Laboratories, Inc., Hercules, CA, USA) read the absorbance at 450 nm after incubation at 37 °C for 2 h. At least three replicates of each group of HCAEC cells were assayed and the results were standardized by subtracting the background reading of the medium from each well.

*1.16 Statistical analysis*

GraphPad Prism 8 (GraphPad Software, La Jolla, CA, USA) was used to perform the statistical analysis and to create the figures. The mean ± SEM were used to express the data and either an unpaired t test or one-way analysis of variance (ANOVA), as appropriate, was used for data analysis. A P value less than 0.05 was considered statistically significant.

**Figure 2**


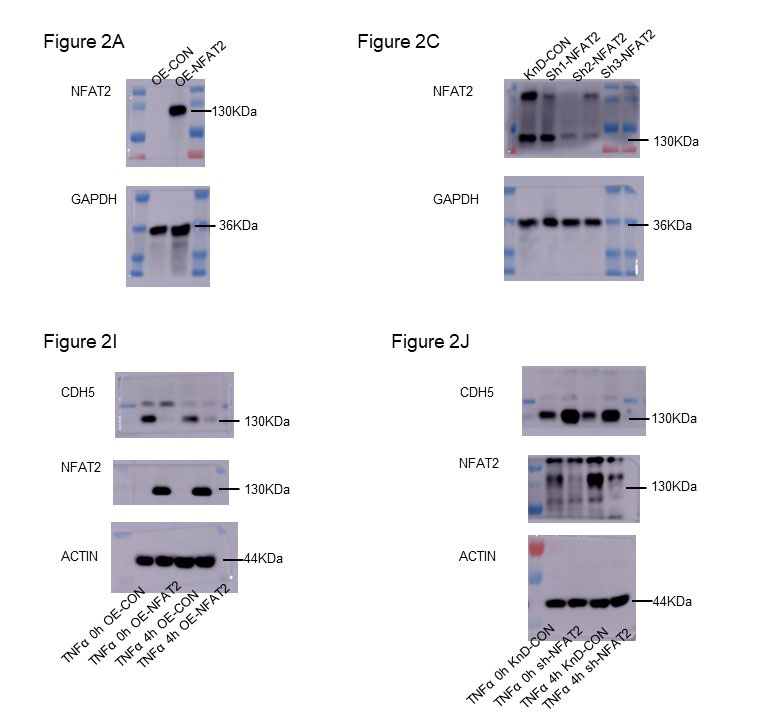


**Figure 3**


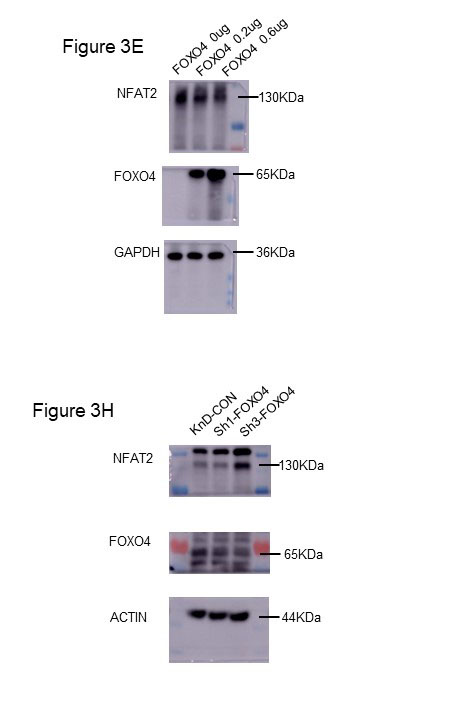


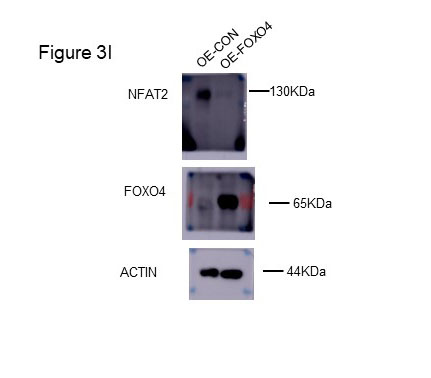


**Figure 4
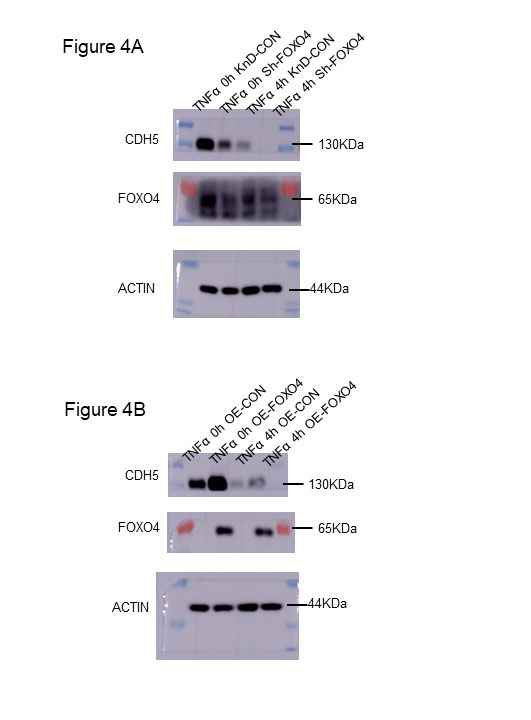
**

**Figure 5**


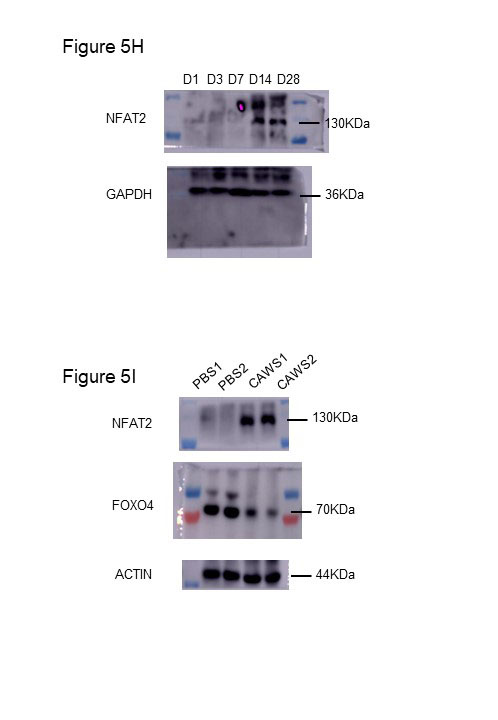


**Figure 6**


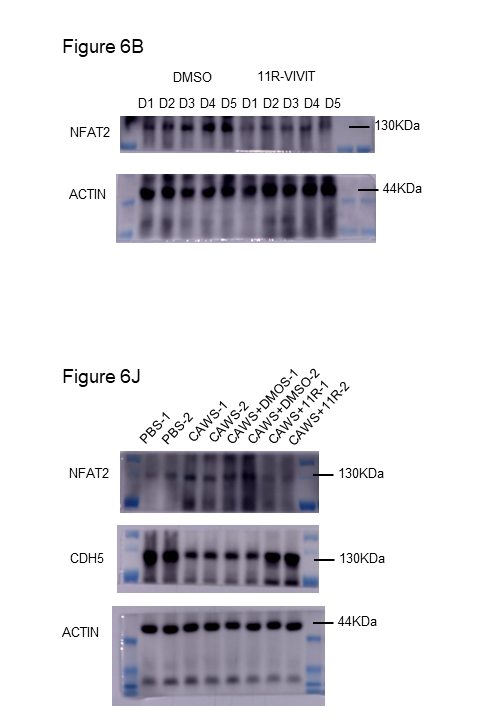


**Figure 7**


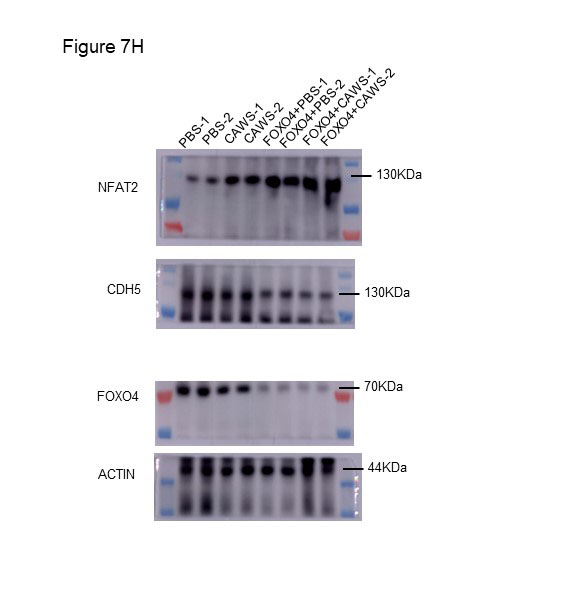


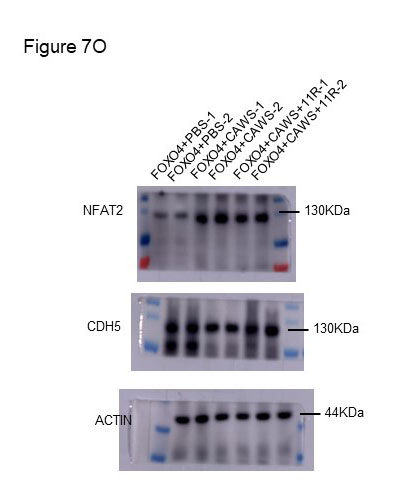


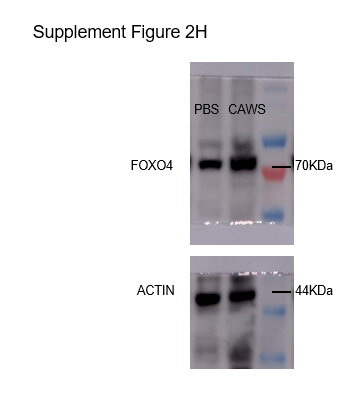

Supplement: Supplementary file 1 [file DataSheet_1.docx]
